# Exploiting deep sulfur conversion by tandem catalysis for all-solid-state lithium–sulfur batteries

**DOI:** 10.1093/nsr/nwaf525

**Published:** 2025-11-21

**Authors:** Huilin Ge, Yu Long, Dulin Huang, Chuannan Geng, Tianran Yan, Haotian Yang, Maoxin Chen, Li Wang, Liang Zhang, Xu Zhang, Zhen Zhou, Chunpeng Yang, Quan-Hong Yang

**Affiliations:** Tianjin Key Laboratory of Advanced Carbon and Electrochemical Energy Storage, State Key Laboratory of Chemical Engineering and Low-Carbon Technology, School of Chemical Engineering and Technology, National Industry-Education Integration Platform of Energy Storage, and Collaborative Innovation Center of Chemical Science and Engineering (Tianjin), Tianjin University, Tianjin 300072, China; Haihe Laboratory of Sustainable Chemical Transformations, Tianjin 300192, China; Tianjin Key Laboratory of Advanced Carbon and Electrochemical Energy Storage, State Key Laboratory of Chemical Engineering and Low-Carbon Technology, School of Chemical Engineering and Technology, National Industry-Education Integration Platform of Energy Storage, and Collaborative Innovation Center of Chemical Science and Engineering (Tianjin), Tianjin University, Tianjin 300072, China; Haihe Laboratory of Sustainable Chemical Transformations, Tianjin 300192, China; Joint School of National University of Singapore and Tianjin University, International Campus of Tianjin University, Fuzhou 350207, China; Department of Chemistry, National University of Singapore, Singapore 117543, Singapore; Interdisciplinary Research Center for Sustainable Energy Science and Engineering (IRC4SE2), School of Chemical Engineering, Zhengzhou University, Zhengzhou 450001, China; Tianjin Key Laboratory of Advanced Carbon and Electrochemical Energy Storage, State Key Laboratory of Chemical Engineering and Low-Carbon Technology, School of Chemical Engineering and Technology, National Industry-Education Integration Platform of Energy Storage, and Collaborative Innovation Center of Chemical Science and Engineering (Tianjin), Tianjin University, Tianjin 300072, China; Haihe Laboratory of Sustainable Chemical Transformations, Tianjin 300192, China; Institute of Functional Nano & Soft Materials (FUNSOM), Jiangsu Key Laboratory of Advanced Negative Carbon Technologies, Soochow University, Suzhou 215123, China; Tianjin Key Laboratory of Advanced Carbon and Electrochemical Energy Storage, State Key Laboratory of Chemical Engineering and Low-Carbon Technology, School of Chemical Engineering and Technology, National Industry-Education Integration Platform of Energy Storage, and Collaborative Innovation Center of Chemical Science and Engineering (Tianjin), Tianjin University, Tianjin 300072, China; Haihe Laboratory of Sustainable Chemical Transformations, Tianjin 300192, China; Joint School of National University of Singapore and Tianjin University, International Campus of Tianjin University, Fuzhou 350207, China; Department of Chemistry, National University of Singapore, Singapore 117543, Singapore; Tianjin Key Laboratory of Advanced Carbon and Electrochemical Energy Storage, State Key Laboratory of Chemical Engineering and Low-Carbon Technology, School of Chemical Engineering and Technology, National Industry-Education Integration Platform of Energy Storage, and Collaborative Innovation Center of Chemical Science and Engineering (Tianjin), Tianjin University, Tianjin 300072, China; Haihe Laboratory of Sustainable Chemical Transformations, Tianjin 300192, China; Joint School of National University of Singapore and Tianjin University, International Campus of Tianjin University, Fuzhou 350207, China; Department of Chemistry, National University of Singapore, Singapore 117543, Singapore; Tianjin Key Laboratory of Advanced Carbon and Electrochemical Energy Storage, State Key Laboratory of Chemical Engineering and Low-Carbon Technology, School of Chemical Engineering and Technology, National Industry-Education Integration Platform of Energy Storage, and Collaborative Innovation Center of Chemical Science and Engineering (Tianjin), Tianjin University, Tianjin 300072, China; Haihe Laboratory of Sustainable Chemical Transformations, Tianjin 300192, China; Institute of Functional Nano & Soft Materials (FUNSOM), Jiangsu Key Laboratory of Advanced Negative Carbon Technologies, Soochow University, Suzhou 215123, China; Interdisciplinary Research Center for Sustainable Energy Science and Engineering (IRC4SE2), School of Chemical Engineering, Zhengzhou University, Zhengzhou 450001, China; Interdisciplinary Research Center for Sustainable Energy Science and Engineering (IRC4SE2), School of Chemical Engineering, Zhengzhou University, Zhengzhou 450001, China; Tianjin Key Laboratory of Advanced Carbon and Electrochemical Energy Storage, State Key Laboratory of Chemical Engineering and Low-Carbon Technology, School of Chemical Engineering and Technology, National Industry-Education Integration Platform of Energy Storage, and Collaborative Innovation Center of Chemical Science and Engineering (Tianjin), Tianjin University, Tianjin 300072, China; Haihe Laboratory of Sustainable Chemical Transformations, Tianjin 300192, China; Tianjin Key Laboratory of Advanced Carbon and Electrochemical Energy Storage, State Key Laboratory of Chemical Engineering and Low-Carbon Technology, School of Chemical Engineering and Technology, National Industry-Education Integration Platform of Energy Storage, and Collaborative Innovation Center of Chemical Science and Engineering (Tianjin), Tianjin University, Tianjin 300072, China; Haihe Laboratory of Sustainable Chemical Transformations, Tianjin 300192, China; Joint School of National University of Singapore and Tianjin University, International Campus of Tianjin University, Fuzhou 350207, China

**Keywords:** all-solid-state batteries, lithium–sulfur batteries, tandem catalysis, Co single atoms, MXene

## Abstract

All-solid-state lithium–sulfur batteries (ASSLSBs) promise high theoretical energy density and inherent safety, but their full capacity delivery is seriously hindered by incomplete sulfur conversion. Here, we propose to exploit deep conversion of S_8_ to Li_2_S via intermediate Li_2_S_2_ by using tandem catalysis for high-capacity ASSLSBs, which we demonstrate by cobalt single-atom catalysts anchored on a conductive MXene substrate. In contrast to commonly believed one-step S_8_ reduction to Li_2_S in ASSLSBs, our results show that tandem catalysis achieves stepwise S_8_ reduction to Li_2_S via Li_2_S_2_, during which atomically dispersed Co sites break S–S bonds and the polar MXene surface facilitates Li^+^ diffusion, significantly reducing the sulfur conversion energy barriers. Consequently, the Co@MX-based ASSLSB reserves a high capacity of 1329 mAh g_S_^−1^ after 2000 cycles at 2.8 mA cm^−2^ at room temperature. This work demonstrates the promise of tandem catalysis for tailoring an all-solid-state sulfur conversion path and exploiting deep sulfur conversion capacity for high-performance ASSLSBs.

## Teaser

This work proposes tandem catalysis to exploit deep conversion of S8 to Li_2_S via intermediate Li_2_S_2_, achieving high-capacity all-solid-state lithiumsulfur batteries and deepening the understanding for solid-state catalytic mechanism.

## INTRODUCTION

All-solid-state lithium–sulfur (Li-S) batteries (ASSLSBs) employing nonflammable inorganic solid electrolytes are considered as a promising next-generation energy-storage system [1–3]. ASSLSBs intrinsically eliminate the notorious shuttle effect of lithium polysulfides in liquid Li-S batteries, and effectively address key safety concerns arising from Li dendrite growth, parasitic reactions between Li metal and organic liquid electrolytes (LEs) and inherent flammability of LE [4–6]. However, ASSLSBs still face critical challenges [2,7–11], particularly the high energy barriers and sluggish redox kinetics of the solid-state S_8_/Li_2_S conversion, which results in low sulfur utilization. Various strategies have been explored to overcome these limitations, including nanosizing S_8_/Li_2_S [12–14], incorporating novel carbon host materials [15–18], using redox mediators [10,19–22] or electrocatalysts [9,23].

**Figure 1.**
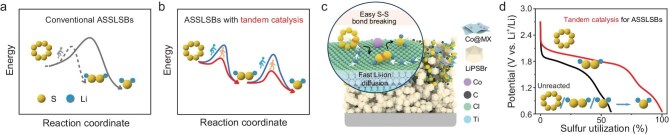
Tandem catalysis for ASSLSBs. (a) Reaction pathways of conventional all-solid-state Li-S batteries. In ASSLSBs, the solid-solid conversion between S_8_ and Li_2_S at constrained three-phase interfaces involves high energy barriers, typically resulting in incomplete sulfur conversion. (b) Reaction pathways of all-solid-state Li-S batteries with tandem catalysis. Tandem catalysis significantly reduces these energy barriers and promotes smoother and more complete reduction of S_8_ to Li_2_S via Li_2_S_2_ intermediates. (c) Schematic tandem catalytic solid-state conversion between S_8_ and Li_2_S on the Co@MX matrix. Co@MX tandem catalyst simultaneously enables facile S–S bond cleavage and rapid Li^+^ migration at an integrated catalytic interface. Specifically, the atomically dispersed Co sites contribute to binding sulfur species and breaking S–S bonds, and the polar surface of MXene is beneficial to fast Li^+^ diffusion. (d) Discharge processes of ASSLSBs with/without tandem catalysis. Tandem catalysis markedly enhances S_8_→Li_2_S_2_→Li_2_S reaction kinetics and sulfur utilization, thereby enabling deep sulfur conversion.

Although considerable progress has been made in improving the capacity and cyclability of ASSLSBs, the all-solid-state S_8_/Li_2_S redox mechanism remains unclear. Earlier studies generally assume that ASSLSBs take a one-step reduction reaction from S_8_ to Li_2_S without forming any intermediates; however, recent evidence reveals the existence of Li_2_S_2_ as an intermediate, and holds that partial S_8_ is finally reduced only to Li_2_S_2_ [9,24,25] (Fig. 1a). Notably, the Li_2_S_2_-to-Li_2_S conversion contributes to half of the theoretical capacity of Li-S batteries [26]. Indeed, the conversion reaction in ASSLSBs is often incomplete, with substantial amounts of Li_2_S_2_ remaining unreacted because of the huge energy barrier. Therefore, it is of critical significance to fully exploit sulfur conversion capacity. Introducing electrocatalysts to lower reaction energy barriers and boost redox kinetics of the sulfur cathode offers a promising solution [27–29]. However, research on catalysis for ASSLSBs is in its infancy and the underlying catalytic mechanism is still poorly understood [30–33]. Some catalysts of liquid Li-S batteries have been directly adopted in ASSLSBs but the critical solid-state catalysis challenges and the fundamental difference from liquid-phase systems are overlooked. In particular, since the solid reactants and solid electrolytes are unable to diffuse freely on the catalyst surface, the adsorbed sulfur species on specific active sites are strongly bound and can hardly desorb and exchange to another site; meanwhile, Li^+^ transport is largely limited, both of which contrast sharply with liquid Li-S catalysis [34,35]. Therefore, rich electroactive sites and fast Li^+^ diffusion on the catalyst surface are indispensable for realizing sufficient sulfur conversion in ASSLSBs.

Herein, we demonstrate a tandem catalysis strategy to segment the reaction pathway, flatten reaction energy barriers and thus realize deep sulfur conversion in ASSLSBs, employing cobalt single atoms (Co SAs) anchored on a conductive MXene matrix (Co@MX) catalyst for ASSLSBs (Fig. 1b, c). In the presence of the tandem catalyst comprising Co SAs and polar MXene, specifically, the atomically dispersed Co sites strongly bind sulfur species to facilitate S–S bond cleavage and the polar Cl-rich MXene surface enables fast Li^+^ diffusion (Fig. 1c), therefore the redox kinetics of stepwise S_8_ reduction to Li_2_S via Li_2_S_2_ intermediates are accelerated. Consequently, the overall conversion rate of S_8_→Li_2_S_2_→Li_2_S is greatly enhanced, and especially the rate-limiting Li_2_S_2_→Li_2_S reduction step is effectively promoted (Fig. 1d). Moreover, abundant Co SA active sites on MXene allow for sustained interaction with more fragmented active material particles during long-term cycling. As a result, the assembled ASSLSB delivers a reversible capacity of 1329 mAh g_S_^–1^ after a remarkable cyclability of 2000 cycles with no capacity decay, even under a high current density of 2.8 mA cm^–2^ at room temperature. This work provides valuable insight into the electrocatalyst design for tailoring the solid-state sulfur redox process, and deepens understanding of solid-state catalytic mechanisms, laying a solid foundation for the practical use of ASSLSBs.

## RESULTS AND DISCUSSION

### Structure of Co@MX and interaction of Li_2_S/Co@MX

**Figure 2.**
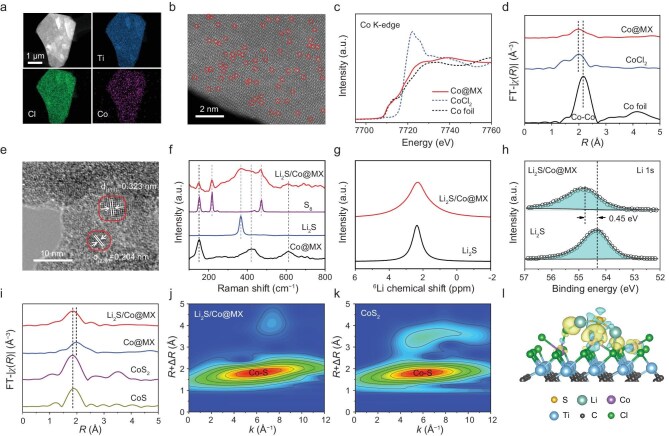
Structure of Co@MX and interaction of Li_2_S/Co@MX. (a) EDS elemental mapping images and (b) HAADF-STEM image of Co@MX. Co SAs are marked by red circles. (c) Co K-edge XANES spectra and (d) corresponding FT-EXAFS spectra of Co@MX, Co foil and CoCl_2_. (e) TEM image of Li_2_S/Co@MX. (f) Raman spectra of Li_2_S/Co@MX, Co@MX, Li_2_S and S_8_. (g) Solid-state ^6^Li MAS-NMR spectra of Li_2_S/Co@MX and Li_2_S. (h) Li 1s XPS spectra of Li_2_S/Co@MX and Li_2_S. (i) FT-EXAFS spectra of Li_2_S/Co@MX, Co@MX, CoS_2_ and CoS. (j and k) Corresponding EXAFS-WT patterns of Li_2_S/Co@MX and CoS_2_. (l) Charge density difference of Li_2_S/Co@MX. Yellow and blue regions represent the accumulation and loss of charge, respectively (isosurface value: 0.018 e Å^−3^).

To construct the tandem catalyst for ASSLSBs, we synthesized Co SAs anchored on Ti_3_C_2_Cl_2_ MXene. Ti_3_AlC_2_ MAX phase was first etched by Lewis acidic molten salts containing CoCl_2_ [36–38], and Co SAs were anchored *in situ* by abundant polar functional groups on the Ti_3_C_2_Cl_2_ MXene substrate after HCl acid pickling (Fig. S1). The energy dispersive X-ray spectroscopy (EDS) elemental mapping images in Fig. 2a depict a uniform distribution of Ti, Cl, and Co elements. Accordingly, the aberration-corrected high-angle annular dark-field scanning transmission electron microscopy (HAADF-STEM) images (Fig. 2b and Fig. S2) clearly show isolated Co SAs (sharp bright dots) uniformly dispersed on the MXene nanosheet. In addition, no detectable Co nanoparticles were observed on the bulk MXene in transmission electron microscopy (TEM) images, which is in agreement with the X-ray diffraction (XRD) results and confirms the existence of Co SAs without nanoparticle (NP) formation (Figs S2 and S3). The Co loading at ∼1 wt% was determined by inductively coupled plasma optical emission spectrometry (ICP-OES).

We used synchrotron-based X-ray absorption spectroscopy (XAS) to further probe the nature of Co SAs [39]. The Co K-edge X-ray absorption near-edge structure (XANES) spectra (Fig. 2c) suggest that the Co valence state in Co@MX is between Co^0^ and Co^2+^. The corresponding Fourier-transformed extended X-ray absorption fine structure (FT-EXAFS) spectrum of Co@MX shows only one prominent peak at a radial distance of 1.98 Å, similar to that of CoCl_2_ (Fig. 2d). The characteristic Co–Co peak at 2.18 Å of metallic Co^0^ is not observed in Co@MX, thus proving the existence of atomically dispersive Co but not Co NPs. Moreover, Co SAs are likely to coordinate with Cl species on MXene via Co–Cl interaction, as supported by the EXAFS wavelet-transform (EXAFS-WT) results (Fig. S4) in which Co@MX and CoCl_2_ show similar maxima in both *k* and *R* space [32].

We obtained a Li_2_S/Co@MX mixture by high-energy ball-milling. Scanning electron microscopy (SEM), high-resolution TEM and EDS elemental mapping images show that Li_2_S and Co@MX are mixed uniformly (Figs S5 and S6). Li_2_S retains its initial crystalline phase, whereas Co@MX undergoes amorphization (Fig. 2e). This is consistent with the XRD pattern with only pronounced diffraction peaks of Li_2_S after mixing (Fig. S7). After ball-milling, minimal amorphous sulfur is detected in the Li_2_S/Co@MX mixture by Raman spectra (Fig. 2f). This phenomenon is probably ascribed to the minor side reaction between Li_2_S and Co@MX, which may be advantageous to improve electrochemical capacity. The solid-state ^6^Li magic-angle-spinning nuclear magnetic resonance (MAS-NMR) spectrum of Li_2_S/Co@MX shows a widened ^6^Li resonance compared with pure Li_2_S (Fig. 2g), indicating a more diverse Li local environment due to the interaction with Co@MX especially Cl-terminated MXene. X-ray photoelectron spectroscopy (XPS) further confirms the chemical interaction between Li_2_S and Co@MX. The Li 1s peak of Li_2_S/Co@MX shifts by 0.45 eV toward higher binding energy (Fig. 2h), which is attributed to the electron-withdrawing effect of surface Cl-groups on the MXene [40]. Such an effect is beneficial to Li_2_S dissociation upon electrochemical oxidation.

The chemical environment change of Co atoms upon interaction with Li_2_S was investigated by XAS. The Co K-edge XANES spectra (Fig. S8) suggest that the valence state of Co in Li_2_S/Co@MX is higher than that in Co@MX but lower than CoS_2_, suggesting partial coordination between Co SAs and S species in Li_2_S. FT-EXAFS results (Fig. 2i) support this inference, as Li_2_S/Co@MX shows a main peak at ∼1.85 Å, which is slightly different from that of Co@MX (1.98 Å) but coincident with the Co-S bond distance in CoS_2_. In addition, EXAFS-WT spectra further prove possible Co–S coordination, as indicated by the approximate maximum in both *k* and *R* space (Fig. 2j, k and Fig. S8). Moreover, the charge density difference calculation (Fig. 2l) illustrates the charge transfer between Li_2_S and Co@MX. The strong interaction between S atoms and Co sites, accompanied with relatively weak Li–Cl bonding plays a key role in promoting the sulfur redox process.

### Reaction pathway of ASSLSBs with tandem catalysis

**Figure 3.**
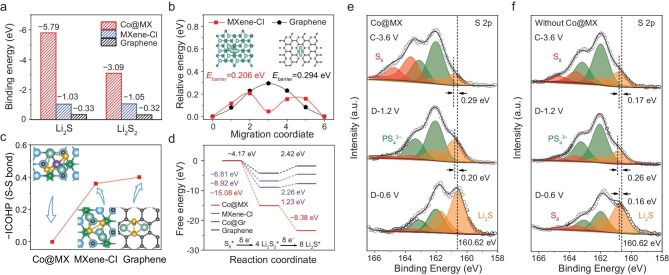
Reaction pathway of ASSLSBs and tandem catalytic mechanism of Co@MX. (a) Binding energies of Li_2_S_2_ and Li_2_S with Co@MX, MXene-Cl and graphene. (b) Energy profiles for Li^+^ diffusion on MXene-Cl and graphene. The insets are the top-view schematic representations of the corresponding Li^+^ diffusion pathways. (c) S–S bond strength derived from integrated COHP (−ICOHP) calculations for Li_2_S_2_ adsorption. The insets are the top-view optimized adsorption conformations of Li_2_S_2_ on Co@MX, MXene-Cl and graphene substrates. (d) Free energy diagram of SRR on Co@MX, MXene-Cl, Co@Gr and graphene. (e and f) *Ex situ* S 2p XPS spectra of the cathodes with/without Co@MX during the first cycle.

Density functional theory (DFT) calculations were performed to acquire an in-depth understanding on the catalytic mechanism of Co@MX in the redox reaction of ASSLSBs [41]. Li_2_S/Li_2_S_2_ molecules show stronger chemical adsorption on Co@MX compared with bare MXene (MXene-Cl) and graphene (Fig. 3a and Fig. S9). The strong interaction between Co@MX and Li_2_S is favorable for weakening Li–S bonds and facilitating Li^+^ dissociation from Li_2_S, which accords well with the above experimental analyses. We also calculated the Li^+^ diffusion barriers on the polar MXene and nonpolar graphene surface. The calculated Li^+^ diffusion barrier along the diffusion coordinate on MXene-Cl is 0.206 eV, lower than that on O-terminated MXene (MXene-O) (0.234 eV) and graphene (0.294 eV) (Fig. 3b and Fig. S10). A lower barrier enhances Li^+^ diffusion and promotes the reaction between lithium and sulfur. The S–S bond strength of adsorbed Li_2_S_2_ was quantitatively evaluated by the crystal orbital Hamilton population (COHP) method. Li_2_S_2_ exhibits a far weaker S–S bond strength on Co@MX than that on MXene-Cl and graphene (Fig. 3c and Fig. S9), suggesting that Co@MX can facilitate S–S bond cleavage and Li_2_S_2_ reduction.

Based on the above analysis, we calculated the free energy landscape of the sulfur reduction reaction (SRR) on different surfaces via intermediate Li_2_S_2_. According to Fig. 3d, the reaction of Li_2_S_2_→Li_2_S shows a large increase of Gibbs free energy, indicating the reduction reaction from Li_2_S_2_ to Li_2_S is the rate-limiting step. The free energy changes on MXene-Cl are lower than those on graphene, consistent with the −ICOHP analysis, highlighting the catalytic potential of MXene-Cl. Furthermore, Co@MX not only boosts S_8_/Li_2_S_2_ conversion with a significantly low free energy change but also renders the Li_2_S_2_→Li_2_S reduction reaction more thermodynamically favorable. In particular, the energetically demanding Li_2_S_2_ reduction reaction becomes a thermodynamically spontaneous reaction with the assistance of Co@MX, allowing for easier Li_2_S nucleation. In addition, the free energy change for each step on Co@MX is distinctly lower than that on the surface of Co SAs supported on graphene (Co@Gr). Hence, the synergistic effect of atomically dispersed Co sites and the polar Cl-rich surface of MXene reveals the essence of the high catalytic activity of Co@MX, collaboratively realizing the tandem catalysis process.

We fabricated ASSLSBs to investigate the tandem catalyzed sulfur conversion in Co@MX-based sulfur cathodes, using an argyrodite-type Li_6_PS_5_X (X = Cl, Br) solid electrolyte, which is a promising candidate due to its high ionic conductivity, favorable mechanical deformation, and acceptable stability against both Li metal and sulfur cathodes [6,42–44] (Figs S11 and S12). XPS was employed to monitor the evolution of sulfur species in the composite cathodes at different charge/discharge states (Fig. 3e, f and Fig. S13). The S 2p spectra reveal the presence of Li_2_S (2p_3/2_ at 160.6 eV) and PS_4_^3−^ (2p_3/2_ at 162.0 eV) in the initial state. After charging to 3.6 V (C-3.6 V, all potentials are vs Li^+^/Li, unless otherwise specified), rich elemental sulfur (S^0^) is detected in the Co@MX-based cathode with minimal residual Li_2_S, while significantly less S^0^ is observed in the cathode without catalyst. After discharging to 1.2 V (D-1.2 V), S^0^ in the Co@MX-based cathode nearly disappears and transforms to Li_2_S_2_/Li_2_S, indicated by a 0.20 eV shift toward higher binding energy in comparison to Li_2_S. Upon further discharging to 0.6 V (D-0.6 V), the Li_2_S peak returns to the initial position and becomes dominant. It can be speculated that S_8_ transforms into Li_2_S_2_ first and Li_2_S_2_ subsequently reduces to Li_2_S. In the absence of Co@MX, residual S^0^ and Li_2_S_2_ are still detectable even at the D-0.6 V state, signifying incomplete sulfur reduction and limited active material utilization. These findings as well as *ex situ* XRD and Raman results (Fig. S14) suggest that Co@MX reduces energy barriers and enhances the reaction rate of each step, thereby realizing deeper S_8_→Li_2_S_2_→Li_2_S conversion. Additionally, time-of-flight secondary ion mass spectroscopy (TOF-SIMS) results also support the S_8_→Li_2_S_2_→Li_2_S conversion pathway by detecting the presence of Li_2_S_2_. Li_2_S_2_ can be effectively differentiated from Li_2_S based on their different mass-to-charge ratios. Figures S15 and S16 shows the mass spectra and spatial distributions of Li_3_S_2_^+^ and Li_3_S^+^ in different cathodes at the D-0.6 V state, where Li_3_S_2_^+^ is reported to more likely originate from Li_2_S_2_ rather than Li_2_S [9], further substantiating the S_8_→Li_2_S_2_→Li_2_S reduction process.

### Reaction kinetics of tandem catalyzed sulfur cathodes

**Figure 4.**
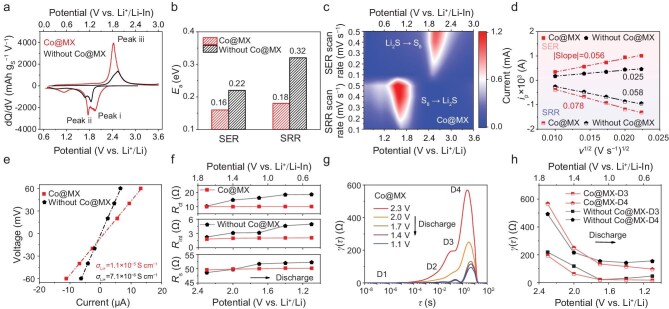
Reaction kinetics of Co@MX-based sulfur cathodes. (a) dQ/dV curves of ASSLSBs with/without Co@MX. (b) Apparent activation energies of the cathodes with/without Co@MX, obtained from CV profiles at different temperatures. (c) Contour plots of CV profiles for the Co@MX-based cathode at different scan rates. (d) Peak current values of CV profiles vs the square root of the scan rates. (e) Effective ionic conductivity of the cathode composites with/without Co@MX, measured by DC polarization. (f) Evolution of *R*_s_, *R*_int_ and *R*_ct_ at different discharge potentials for ASSLSBs with/without Co@MX, revealed by *in situ* EIS measurements. (g) DRT curves calculated from *in situ* EIS measurements during the discharge process of Co@MX-based ASSLSBs. (h) Relaxation-based function γ(τ) obtained from DRT spectra for the discharge process of ASSLSBs with/without Co@MX.

To elucidate the impact of tandem catalysis, we systematically investigated the electrochemical behaviors and analyzed the redox kinetics of Co@MX-based sulfur cathodes. The ASSLSB without Co@MX exhibits much lower capacities and a more severe polarization in the galvanostatic charge–discharge profiles, as further reflected by the corresponding differential capacity vs voltage (dQ/dV) curves (Fig. 4a and Fig. S17). The split in the reduction peak (labelled as peaks i and ii) in Fig. 4a is noticed in both cathodes, implying a two-step reduction involving the formation of Li_2_S_2_ intermediates followed by the formation of Li_2_S [11,24,35]. The apparent activation energies (*E*_a_) of the sulfur redox reaction were determined from cyclic voltammetry (CV) profiles at different temperatures [29]. A linear relationship was observed between the logarithmic value of the peak current and the inverse of the absolute temperature for both cathodes, focusing on the main redox peaks (Fig. S18). The calculated *E*_a_ values of Co@MX-based cathodes are much lower than those without Co@MX, representing lower reaction energy barriers (Fig. 4b). In addition, galvanostatic intermittent titration technique (GITT) measurement analysis shows a higher amount of electrochemically active sulfur in Co@MX-based cathodes (Fig. S19). The overpotential gradually increases with the deepening of charge/discharge, showing that the second de-lithiation/lithiation step (Li_2_S_2_→S_8_/Li_2_S_2_→Li_2_S) is more kinetically difficult [10].

We further investigated how the Co@MX tandem catalyst influences ion transport in solid-state sulfur cathodes during the sulfur redox process. A linear relationship between CV peak current and the square root of the scan rates is observed for both cathodes; however, the Co@MX-based cathode exhibits higher current responses and steeper slopes, suggesting enhanced Li^+^ diffusion (Fig. 4c, d and Fig. S20). The composite with Co@MX exhibits substantially lower ionic resistance than the counterpart without Co@MX measured by direct-current (DC) polarization techniques, as determined by Ohm’s law (Fig. 4e and Fig. S21). Correspondingly, the effective ionic conductivity of Co@MX-based composite is significantly improved, up to 1.1 × 10^−5^ S cm^−1^. These results highlight the key role of Co@MX, especially the Cl-rich polar surface of MXene, in promoting Li^+^ diffusion and enhancing Li^+^ migration across the Li_2_S–Li_6_PS_5_Br (LiPSBr) interface. The system resistance (*R*_s_), interface resistance (*R*_int_), and charge transfer resistance (*R*_ct_) at various charge/discharge potentials [40,45], were also extracted and compared by fitting *in situ* electrochemical impedance spectra (EIS) of ASSLSBs (Fig. 4f, Fig. S22 and Tables S1 and S2). Both *R*_ct_ and *R*_int_ in the Co@MX-based cathode are lower and more stable, which is attributed to the enhanced Li^+^/e^−^ diffusion enabled by Co@MX and the structural integrity provided by the robust MXene matrix, which helps maintain an intimate cathode/electrolyte interface. We distinguished the resistance evolution of each part in the timescale through the distribution of relaxation time (DRT) analysis [46]. As shown in Fig. 4g, the D3 peak (relaxation time: 0.1–1 s) is related to the ion transport resistance across the cathode interface, while the D4 peak, with the largest time constant, is assigned to the cathode charge transfer process [47]. For both charge and discharge processes, the intensities of these peaks almost remain lower in the Co@MX-based cathode than those without Co@MX (Fig. 4g, h and Fig. S23). These DRT results elaborate that Co@MX enhances charge transfer in the composite cathode and facilitates Li^+^ transport across the cathode/electrolyte interface, consistent with the EIS analysis.

### All-solid-state Li-S battery performances

**Figure 5.**
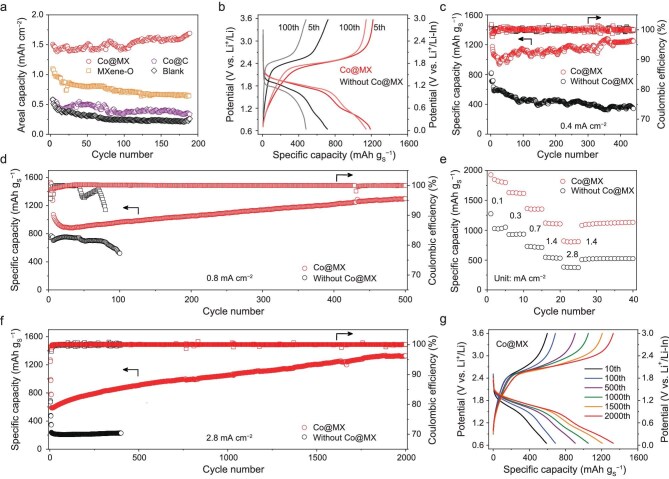
Electrochemical performance of ASSLSBs at room temperature. (a) Cycling performance of ASSLSBs using different catalysts with a Li_2_S loading of 2.4 mg cm^−2^ at 0.2 mA cm^−2^. (b) Galvanostatic charge–discharge profiles and (c) cycling performance of ASSLSBs with/without Co@MX with a Li_2_S loading of 1.1 mg cm^−2^ at 0.4 mA cm^−2^. The batteries were preconditioned through several cycles at lower current densities. (d) Cycling performance of ASSLSBs with/without Co@MX with a Li_2_S loading of 1.1 mg cm^−2^ at 0.8 mA cm^−2^. (e) Rate performance of ASSLSBs with/without Co@MX with a Li_2_S loading of 1.2 mg cm^−2^. (f) Cycling performance and (g) galvanostatic charge–discharge profiles of different cycles for Co@MX-based ASSLSBs with a Li_2_S loading of 1.2 mg cm^−2^ at 2.8 mA cm^−2^.

To validate the unique effect of Co@MX for ASSLSBs, we systematically compared battery performances using different catalysts. The Co@MX-based Li-S battery exhibits a much higher discharge capacity and cycling stability than those employing conventional MXene-O without Co, or Co SAs supported on porous carbon (Co@C) or supported on O-rich MXene (Fig. 5a, Figs S24–S28 and Tables S3 and S4). These results corroborate the cooperative effect of the Co@MX tandem catalyst, in agreement with theoretical calculations (Fig. 3a–d and Fig. S10). Due to Co SAs active sites binding sulfur species and breaking S–S bonds and subsequently polar MXene-Cl surface facilitating Li^+^ diffusion, the reaction kinetics are accelerated and thereby sulfur utilization is improved. The optimal 4:2 mass ratio of Li_2_S to Co@MX is determined based on the optimized composition of the cathode (Fig. S29). The ASSLSB with Co@MX delivers an initial discharge capacity of 1176 mAh g_S_^−1^ at 0.4 mA cm^−2^, significantly outperforming the one without Co@MX (704 mAh g_S_^−1^) (Fig. 5b). After 100 cycles, the Co@MX-based cathode retains a discharge capacity of 1129 mAh g_S_^−1^, while the cathode without Co@MX decreases to 471 mAh g_S_^−1^, due to the large S_8_→Li_2_S_2_→Li_2_S conversion barrier and incomplete conversion reaction. Notably, the Co@MX-based battery retains excellent capacity of 1249 mAh g_S_^−1^ for over 440 cycles (Fig. 5c). Even at a higher current density of 0.8 mA cm^−2^, the Co@MX-based cathode delivers an initial reversible capacity of 1071 mAh g_S_^−1^ and shows no capacity decay over 500 cycles (Fig. 5d). In contrast, the cathode without catalyst exhibits a lower capacity with a rapid decay and poor reversibility within 100 cycles. When increasing the active material loading to 2.6 mg cm^−2^, the ASSLSB with Co@MX can be cycled for >440 cycles still with no capacity decay (Fig. S30).

Due to the tandem catalytic effect, the Co@MX-based cathode shows superior rate performance compared to that without Co@MX (Fig. 5e). The cathode with Co@MX delivers almost twice the discharge capacity than that without a catalyst under various current densities ranging from 0.1 to 2.8 mA cm^−2^. Remarkably, the Co@MX-based cathode retains an outstanding capacity of 1329 mAh g_S_^−1^ after 2000 cycles at a high current density of 2.8 mA cm^−2^ (Fig. 5f, g), while the capacity of the cathode without catalyst remains below 300 mAh g_S_^−1^. The high-loading cathode with Co@MX delivers areal capacities of ∼2 mAh cm^−2^ and 3.8 mAh cm^−2^ after 100 cycles for Li_2_S and S_8_ cathodes, respectively, showing excellent practical feasibility (Fig. S31). These results highlight that Co@MX acting as a tandem catalyst accelerates Li_2_S/S_8_ redox kinetics and enables ASSLSBs with high-rate, high-loading and long-time cycling.

Interestingly, the capacities of Co@MX-based cathodes gradually increase during cycling, eventually surpassing its initial capacity, particularly under high current densities (Fig. 5c, d and f). Undoubtedly, reversible lithiation/delithiation of LiPSBr electrolytes or Co@MX could have caused some extra capacities [48], but this limited contribution cannot explain the continuous growth in capacity (Fig. S32). More importantly, the above capacity increasing behavior can be elucidated by the unique catalytic mechanism in the all-solid-state system from both microscopic and molecular perspectives. At the microscale level, the inner cores of initially micron-sized Li_2_S particles are electrochemically inert, resulting in a low initial capacity. Moreover, these large particles experience huge volume change and severe pulverization during cycling, leading to fragmentation, detachment with ionic/electronic conductors, and thus accumulation of dead S_8_/Li_2_S [49]. The Co@MX matrix, however, mitigates contact loss and allows for efficient Li^+^ diffusion and high e^−^ conduction [50], thus enabling full conversion and maximized utilization of the fragmented active material particles, as shown in the SEM images of the cathodes before and after cycling (Figs S33–S35). At the molecular scale, for solid-state catalysis, the adsorbed sulfur species on specific active sites are almost fixed and rarely undergo site desorption, and the solid electrolytes are also immobile causing limited Li^+^ transport, which is quite different from liquid Li-S catalysis [34]. Hence, the rich surface-active sites and fast Li^+^ surface diffusion of Co@MX play a significant role. The utilization of Co@MX catalytic sites improves progressively over prolonged cycling, as fragmented active material particles increasingly contact and interact with Co SA active sites, which contributes to the increase in battery capacity as was verified by the post-mortem XPS and DRT analysis (Figs S36 and S37).

To confirm the critical role of Co SAs in enhancing electrochemical performance, we explored and compared a series of MXene samples with varying Co loadings (Fig. S38). With an increase of the Co content, Co SAs tend to aggregate into nanoparticles, resulting in lower discharge capacities in comparison to Co@MX. These findings highlight that the abundant atomically dispersed Co active sites on the polar MXene surface allows for efficient tandem catalysis with more reactive sulfur species during extended cycling compared to Co NPs, which is an indispensable factor for effective catalysis.

## CONCLUSION

In summary, we employed a tandem catalysis strategy, typically demonstrated by Co SAs anchored on MXene nanosheets, to facilitate deep S_8_ reduction to Li_2_S via Li_2_S_2_ intermediates, thereby exploiting the sulfur conversion capacity of ASSLSBs. Theoretical calculations and experimental analyses confirm that the intense chemical interaction between sulfur species and both atomically dispersed Co sites and polar Cl-groups of MXene, combined with rapid Li^+^ surface diffusion, collectively boosts sulfur redox reactions. The Co@MX tandem catalyst endows all-solid-state sulfur cathodes with rich active sites and fast electronic/ionic conduction. It accelerates the key step of Li_2_S_2_-to-Li_2_S conversion during discharge and synergistically decreases the energy barrier of each reaction step, thus achieving deep sulfur conversion. Furthermore, high-density Co SA sites on MXene ensure an increasing tandem catalytic effect with fragmented active material particles over prolonged cycling. The resulting ASSLSB with Co@MX delivers a reversible capacity of 1329 mAh g_S_^−1^ after 2000 cycles at 2.8 mA cm^−2^, and the high-loading cathode maintains an areal capacity of 3.8 mAh cm^−2^ after 100 cycles at room temperature. Our work demonstrates that tandem catalysis can regulate the reaction pathway for the all-solid-state sulfur redox process in order to realize high-capacity and long-life Li-S batteries, and holds great potential to propel the advancement of ASSLSBs.

## METHODS

### Synthesis of Co@MX

The Co@MX was synthesized by etching Ti_3_AlC_2_ MAX phase in a mixture of CoCl_2_, NaCl and KCl molten salts at 700°C for 1 h in an argon (Ar) atmosphere. The resulting solid residue was immersed and stirred in HCl solution (9 M) for 24 h to remove Co particles. After several centrifugation–rinsing cycles with deionized (DI) water, the product was dried at 60°C for 12 h. The Co content in MXene was regulated by controlling the concentration of HCl solution during the acid pickling process. Specifically, the MXene samples with higher Co loadings were synthesized using the same method except for decreasing the HCl concentration to 0.5 M and replacing HCl with DI water, respectively.

### Synthesis of LiPSBr electrolyte

LiPSBr was typically synthesized by ball milling Li_2_S, LiBr and P_2_S_5_ with a stoichiometric molar ratio at 550 rpm for 10 h in an Ar atmosphere. After that, the mixtures were pressed into pellets and annealed at 550°C for 10 h in a quartz tube under vacuum.

### Preparation of composite cathodes

The Li_2_S/Co@MX was prepared by simply ball milling Li_2_S and Co@MX with an optimum mass ratio of 4:2 at 550 rpm for 5 h in an Ar atmosphere. The Li_2_S/Co@MX composite cathode was prepared by ball milling the above Li_2_S/Co@MX, Ketjen black (KB) and LiPSBr with a mass ratio of 6:2:4 at 550 rpm for 5 h in an Ar atmosphere. The same procedure was used to prepare the cathode without Co@MX with a mass ratio of 4:2:4 corresponding to Li_2_S:KB:LiPSBr. The KB-LiPSBr and Co@MX-KB-LiPSBr cathodes were similarly prepared by ball milling only KB and LiPSBr with a mass ratio of 2:4, and Co@MX, KB and LiPSBr with a mass ratio of 2:2:4, respectively.

### Assembly of ASSLSBs

The ASSLSBs were assembled in model cells with a diameter of 10 mm by a cold pressing method inside an Ar-filled glovebox. Specifically, 90 mg of LiPSBr powders were first pressed at 250 MPa for 3 min in the cell to form a pellet. Next, composite cathode powders of the desired amount were uniformly spread on the surface of the electrolyte pellet, and pressed at 250 MPa for further 3 min. Then a Li-In alloy was placed on the other side of the pellet at 125 MPa for 3 min. Finally, the cells were compressed by three bolts. The effective ionic conductivities of the cathode composites with/without Co@MX were measured using an electron-blocking symmetric cell.

## Supplementary Material

nwaf525_Supplemental_Files
