# Supplementary material for: Exploiting deep sulfur conversion by tandem catalysis for all-solid-state lithium–sulfur batteries
Source: Natl Sci Rev. 2025 Nov 21;13(1):nwaf525. doi: 10.1093/nsr/nwaf525 (PMC12796810; doi:10.1093/nsr/nwaf525)
Supplement: nwaf525_Supplemental_Files [file nwaf525_supplemental_files.zip › Supplementary Data.pdf]

## Supplementary Information

# Exploiting deep sulfur conversion by tandem catalysis for all-solid-state lithium–sulfur batteries

Huilin Ge[1,6,†], Yu Long[1,2,3,6,†], Dulin Huang[4,†], Chuannan Geng[1,6], Tianran Yan[5], Haotian Yang[1,2,3,6], Maoxin Chen[1,2,3,6], Li Wang[1,6], Liang Zhang[5,*], Xu Zhang[4,*], Zhen Zhou[4], Chunpeng Yang[1,6,*] and Quan-Hong Yang[1,2,6,*]

[1]Nanoyang Group, Tianjin Key Laboratory of Advanced Carbon and Electrochemical Energy Storage, State Key Laboratory of Chemical Engineering and Low-Carbon Technology, School of Chemical Engineering and Technology, National Industry-Education Integration Platform of Energy Storage, and Collaborative Innovation Center of Chemical Science and Engineering (Tianjin), Tianjin University, Tianjin 300072, China;

[2]Joint School of National University of Singapore and Tianjin University, International Campus of Tianjin University, Fuzhou 350207, China;

[3]Department of Chemistry, National University of Singapore, Singapore 117543, Singapore;

[4]Interdisciplinary Research Center for Sustainable Energy Science and Engineering (IRC4SE2), School of Chemical Engineering, Zhengzhou University, Zhengzhou 450001, China;

[5]Institute of Functional Nano & Soft Materials (FUNSOM), Jiangsu Key Laboratory of Advanced Negative Carbon Technologies, Soochow University, Suzhou 215123, China;

[6]Haihe Laboratory of Sustainable Chemical Transformations, Tianjin 300192, China

***Corresponding authors.** E-mails: liangzhang2019@suda.edu.cn; zzuzhangxu@zzu.edu.cn; cpyang@tju.edu.cn; qhyangcn@tju.edu.cn

[†]Equally contributed to this work.

## Methods

### Synthesis of MXene-O

The MXene-O was synthesized by etching $Ti_3AlC_2$ powders in the mixture solution of HCl and LiF. After stirring for 30 h, the etched products were washed with DI water and centrifuged for several times until the pH of the supernatant approached 6. The precipitation was mixed with DI water and sonicated for 1 h, while bubbling Ar gas through the mixture. After centrifuging for 1 h, the $Ti_3C_2T_x$ colloidal solution was obtained by collecting the supernatant. Finally, MXene-O was obtained by freeze-drying the colloidal solution.

### Preparation of composite cathodes

The $S_8$/KB was prepared by a simple melt-diffusion method. The powders with a mass ratio of 8: 2 were hand ground and heated at 155 °C for 12 h. The obtained $S_8$/KB and Co@MX were ball milled with mass ratio of 5: 2 at 550 rpm for 5 h. The $S_8$/Co@MX composite cathode was prepared by ball milling the above $S_8$/KB-Co@MX, carbon nanotubes (CNT) and LiPSBr at 550 rpm for 5 h in Ar atmosphere and the mass ratio of $S_8$, Co@MX, carbon and LiPSBr was controlled to 0.24: 0.12: 0.16: 0.48.

### Material characterization

The morphology and structure images were taken from SEM (Regulus 8100, Hitachi), TEM (JEM-2100F, JEOL) equipped with EDS system (Oxford) and HAADF-STEM (JEM-ARM300F2, JEOL) equipped with spherical probe aberration corrector. The crystal structures were characterized by X-ray diffractometer (SmartLab, Rigaku) with Cu Kα radiation (λ=0.154 nm). Raman spectra were collected on Raman spectrometer (HORIBA Scientific LabRAM HR) with a 532 nm laser. ICP-OES analysis was conducted on Agilent 5110 instrument. The solid-state $^6$Li MAS-NMR spectra were record on JEOL JNM ECZ600R at a spinning speed of 15 kHz. The investigation of chemical state was carried out on XPS (K-Alpha$^+$, Thermo Fisher Scientific) and the binding energies were calibrated by setting the measured binding energy of

C 1s to 284.8 eV. The Co K-edge XAFS spectra were performed at the BL11B hard X-ray beamline station in the Shanghai Synchrotron Radiation Facility (SSRF). TOF-SIMS spectra were collected on PHI nanoTOF II Time-of-Flight SIMS with a pulsed 30 keV $Bi_3^+$ primary cluster ion beam to generate secondary ions.

**Electrochemical testing of ASSLSBs**

The galvanostatic charge–discharge measurements were conducted using Landt (CT3002A5V2mA&20mA8C1U, Wuhan, China) and NEWARE battery test system (CT-4008Tn-5V10mA-164, Shenzhen, China). The ASSLSBs were tested in the range of 0.6–3.6 V under ambient air. For GITT measurements, the cells were tested at a current pulse of 0.08 A $g^{-1}$ for 30 min followed by a 4 h relaxation. CV curves were obtained using Princeton workstation at different scan rates from 0.1 to 0.5 mV $s^{-1}$. To determine $E_a$ of $Li_2S/S_8$ redox process, the peak currents of ASSLSBs with/without Co@MX at various temperatures were measured. The $E_a$ values were calculated based on the temperature-dependent peak current ($i_p$) according to the Arrhenius equation [1]:

$$i_p \propto k = A \times e^{-\frac{E_a}{RT}}$$

where $k$ is the rate constant, $A$ is the pre-exponential factor, $R$ is the universal gas constant and $T$ is the absolute temperature (in Kelvin). DC polarization measurements were performed on BioLogic workstation by applying a sequence of potentials of −60, −40, −20, +20, +40 and +60 mV with 2 h equilibration time at each potential. EIS spectra were recorded on BioLogic workstation in a frequency range of 1 MHz to 0.1 Hz with an amplitude of 10 mV. The corresponding DRT analysis was performed to reflect the resistance evolution of each part in the timescale.

**Computational method**

All the computations were carried out by the DFT method including van der Waals (vdW) corrections, as implemented in the Vienna ab initio Simulation Package (VASP) [2]. The

Perdew-Burke-Ernzerhof (PBE) functional within the generalized gradient approximation (GGA) was used to describe the exchange-correlation interaction [3]. Projector augmented wave (PAW) methods were used for pseudopotentials [4]. An energy cutoff of 500 eV was adopted for the plane-wave basis. The vacuum layers were configured to exceed 15 Å, ensuring the effective decoupling of interactions between periodic images. The Brillouin zones were sampled using Gamma-centered k-mesh of 3 × 3 × 1. The energy convergence criterion of geometry relaxation was set to $10^{-5}$ eV. The rest atomic layers and adsorbates were free to relax until the net force per atom is less than 0.05 eV $Å^{-1}$. The DFT-D3 method was used to describe the van der Waals interaction [5]. The VASPKIT code was used for the post-processing of the VASP computational data [6]. The structures were visualized using the VESTA package [7]. The differential charge density was calculated according to: $\Delta\rho = \rho_{AB} - \rho_{A} - \rho_{B}$, where $\rho_{AB}$, $\rho_{A}$ and $\rho_{B}$ represent the total charge density, the substrate charge density, and the surface adsorbate charge density, respectively. The adsorption energy was calculated according to the equation: $E_{ads} = E_{AB} - E_{A} - E_{B}$, where $E_{AB}$ is the energy of optimized structures for molecules adsorbed on the planes, $E_{A}$ is the energy of isolated molecules, and $E_{B}$ is the energy of the optimized structures of the substrate models. The calculation of the free energy for the SRR steps in the diagram is based on: $4^* + S_8 + 8Li^+ + 8e^- \rightarrow 4Li_2S_2^*$ and $4Li_2S_2^* + 8Li^+ + 8e^- \rightarrow 4Li_2S^* + 4Li_2S$. The Gibbs free energy change of each molecule ($G$) was calculated as: $G = E_{DFT} + E_{ZPE} - TS$. where $E_{DFT}$ and $TS$ are the zero-point energy correction and the entropy change at room temperature (298.15 K). The ($E_{ZPE} - TS$) term is calculated by the VASPKIT package [6]. The Gibbs free energy change for each elemental step ($\Delta G_i$) was calculated by equations: $\Delta G_1 = 4G_{Li2S2*} - 8G_{Li+} - G_{S8} - 4G_{*} + neU$ and $\Delta G_2 = 4G_{Li2S*} + 4G_{Li2S} - 8G_{Li+} - 4G_{Li2S2*} + neU$, where n is the number of electrons involved in the electrochemical reaction, e is the elementary charge, and $U$ is the electrode potential. The crystal orbital Hamilton populations (COHP) calculations were analyzed by the LOBSTER package [8]. An improved climbing image

nudged elastic band (CI-NEB) method, based on constrained internal coordinates and energy-based techniques, was implemented in VASP to investigate the energy barriers for ion diffusion on different surfaces [9].

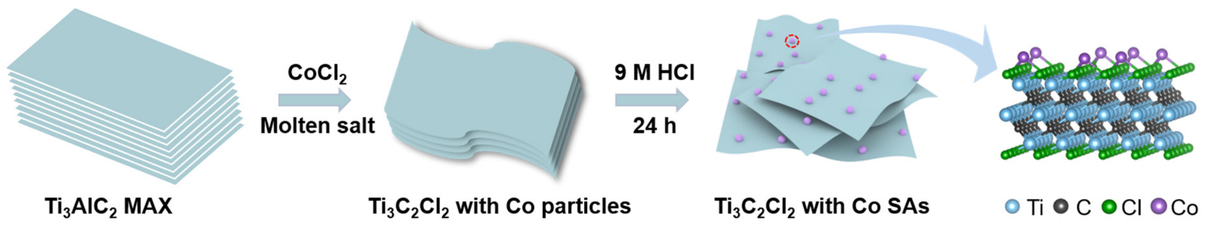


**Fig. S1** Schematic preparation process and crystal structure of Co@MX. $Ti_3AlC_2$ MAX phase was firstly etched by molten salts containing $CoCl_2$ and followingly soaked in HCl acid solution to remove Co particles. The residual Co in $Ti_3C_2Cl_2$ *in situ* form Co SAs.

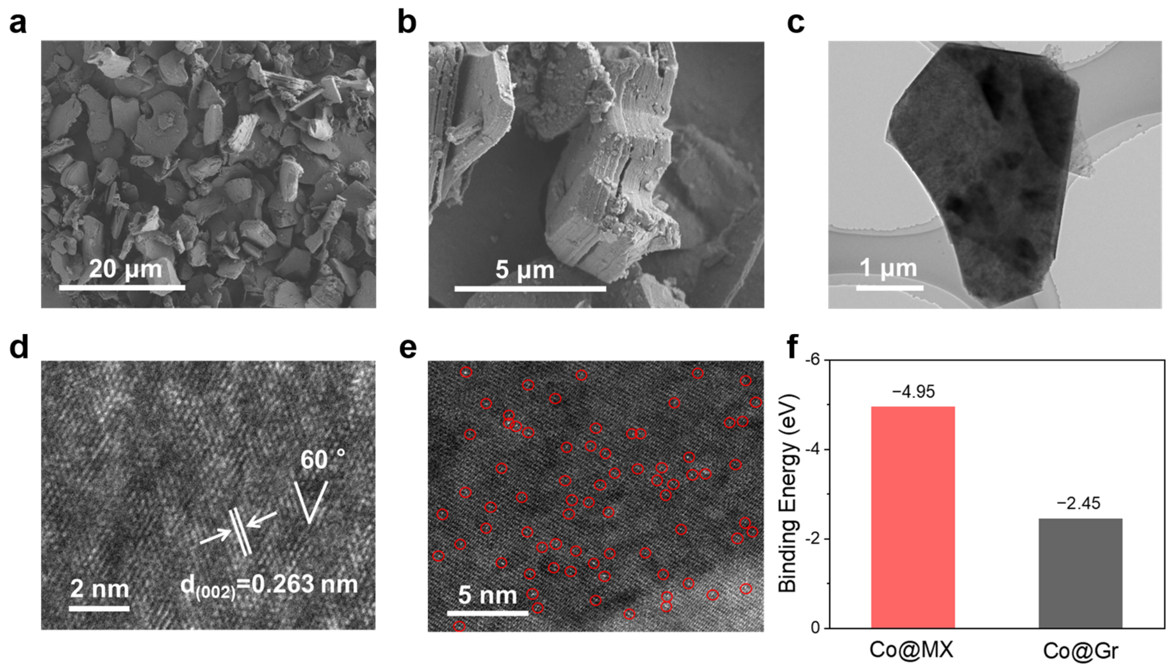


**Fig. S2** (a and b) SEM images and (c and d) TEM images and (e) HAADF-STEM image of Co@MX. SEM images reveal that Co@MX presents multilayer flakes. The crystalline nature of MXene substrate is validated by high-resolution TEM image. (f) Stability comparison for binding Co SAs between MXene-Cl and graphene. DFT calculated binding energy of Co@MX is lower than that of Co@Gr, demonstrating that MXene-Cl shows better stability for binding Co SAs.

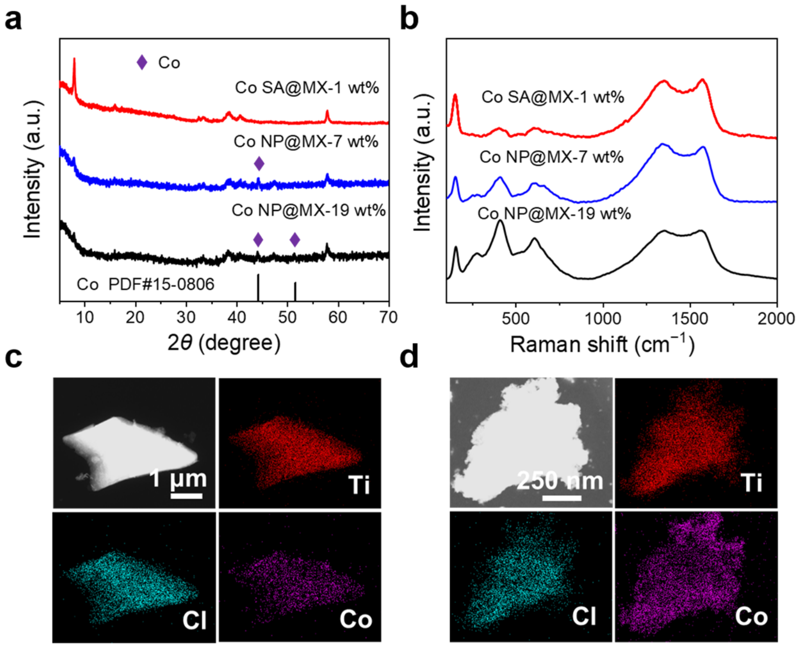


**Fig. S3** (a) XRD patterns and (b) Raman spectra of MXene with different contents of Co. The Co SAs transform into Co NPs with characteristic XRD peaks by decreasing HCl concentration during the preparation process. (c and d) EDS elemental mapping images of Co NP@MX-7 wt% and Co NP@MX-19 wt%.

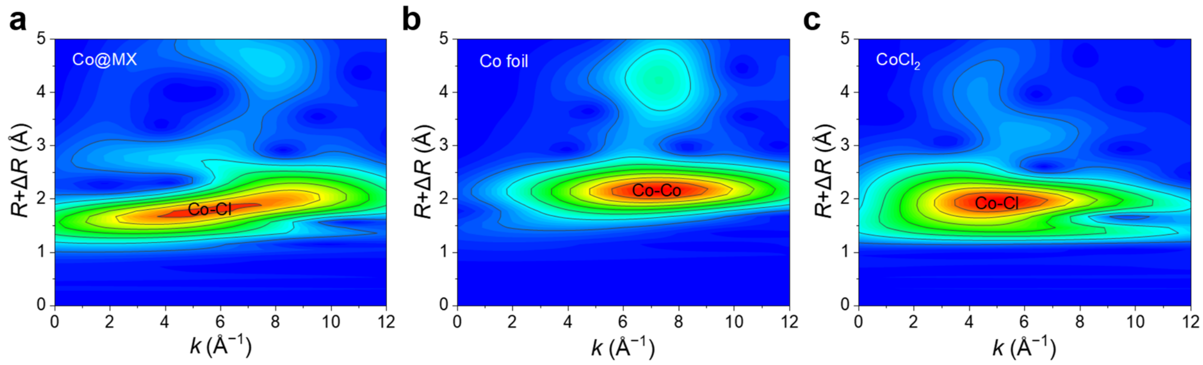


**Fig. S4** (a–c) EXAFS-WT patterns of Co@MX, Co foil and $CoCl_2$.

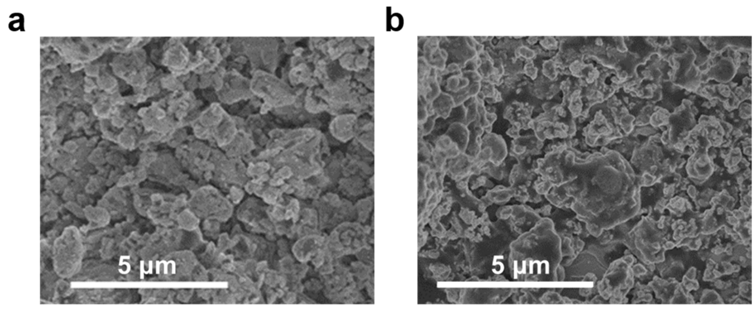


**Fig. S5** (a and b) SEM images of $Li_2S$ and $Li_2S$/Co@MX.

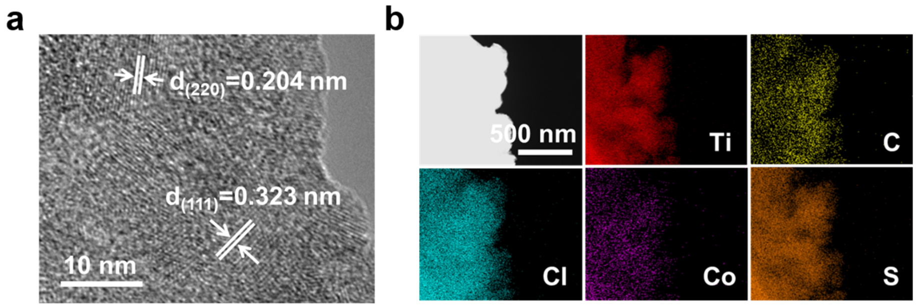


**Fig. S6** (a) TEM image and (b) EDS elemental mapping images of $Li_2S$/Co@MX. Two lattice planes (111) and (220) of $Li_2S$ can been clearly observed.

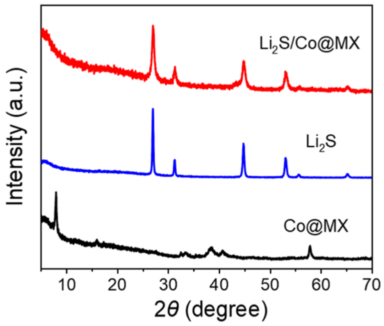


**Fig. S7** XRD patterns of $Li_2S$/Co@MX, Co@MX and $Li_2S$.

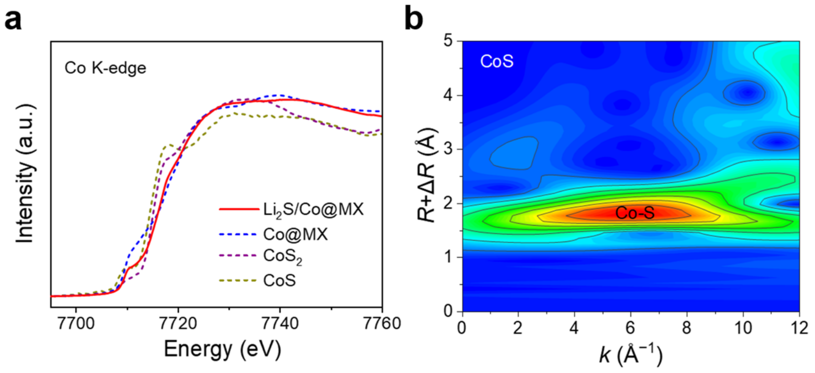


**Fig. S8** (a) Co K-edge XANES spectra of $Li_2S$/Co@MX, Co@MX, $CoS_2$ and CoS. (b) Corresponding EXAFS-WT pattern of CoS.

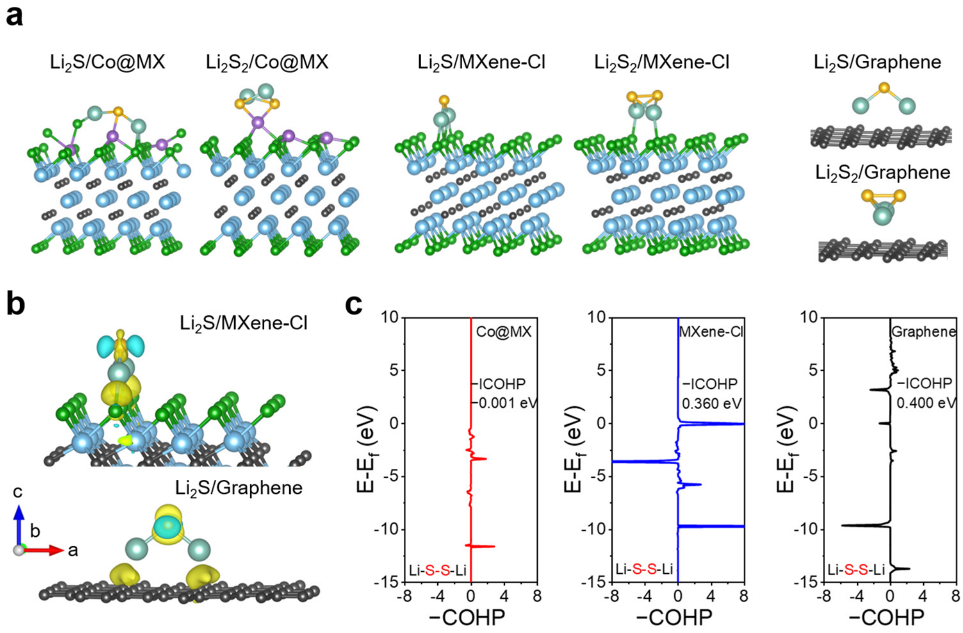


**Fig. S9** (a) The optimized adsorption conformations of $Li_2S_2$ and $Li_2S$ on Co@MX, MXene-Cl and graphene substrates. (b) The charge density difference of $Li_2S$/MXene-Cl and $Li_2S$/graphene (isosurface value: 0.018 e Å$^{-3}$). (c) COHP of S-S bond for $Li_2S_2$ adsorption on Co@MX, MXene-Cl and graphene.

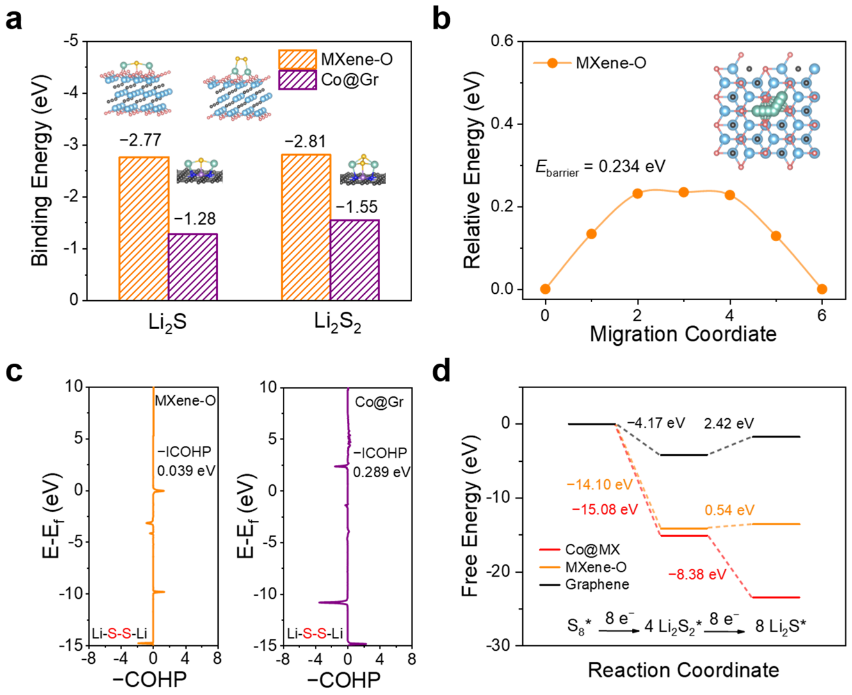


**Fig. S10** (a) Binding energies of $Li_2S_2$ and $Li_2S$ with MXene-O and Co@Gr. The insets are the optimized adsorption conformations. (b) The energy profile for $Li^+$ diffusion on MXene-O. The inset is the top-view schematic representation of the corresponding $Li^+$ diffusion pathway. (c) S-S bond strength derived from integrated COHP (−ICOHP) calculations for $Li_2S_2$ adsorption. (d) Free energy diagram of SRR on Co@MX, MXene-O and graphene.

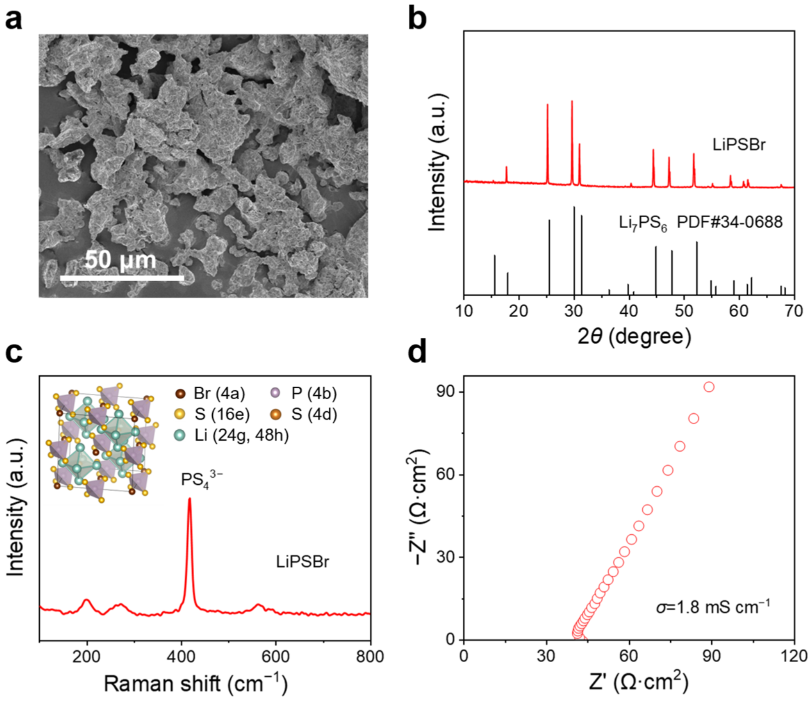


**Fig. S11** (a) SEM image and (b) XRD pattern of LiPSBr. (c) Raman spectrum of LiPSBr. The inset is the schematic crystal structure of LiPSBr. (d) Nyquist plot of LiPSBr. The calculated ionic conductivity at room temperature is 1.8 mS $cm^{-1}$.

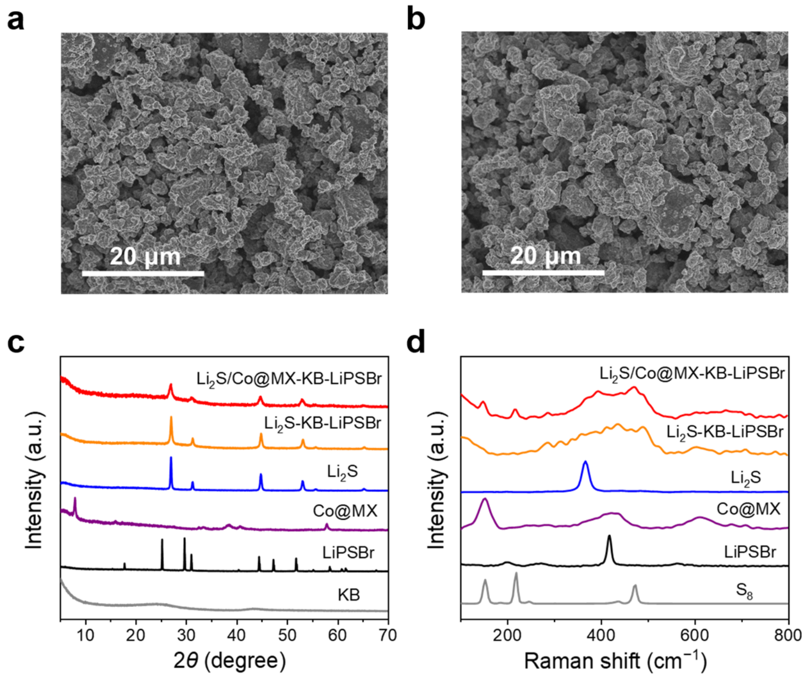


**Fig. S12** (a and b) SEM images of the composite cathodes with Co@MX and without Co@MX. (c) XRD patterns and (d) Raman spectra of the composite cathodes with/without Co@MX.

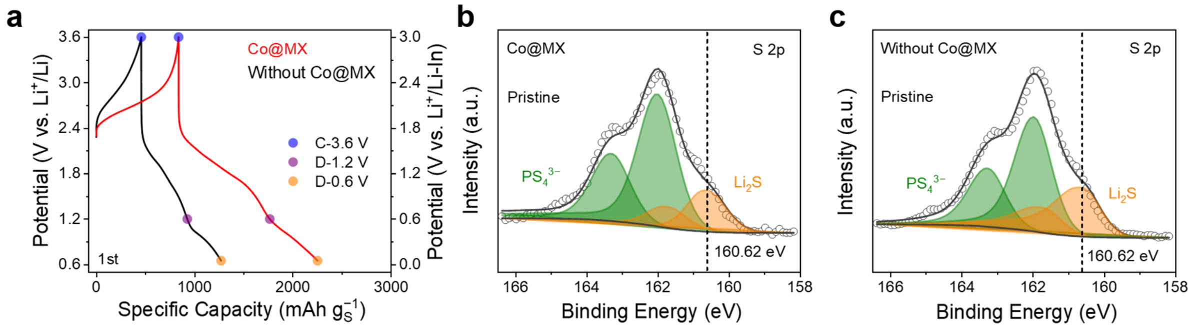


**Fig. S13** (a) Galvanostatic charge–discharge profiles comprising different states: C-3.6 V, D-1.2 V and D-0.6 V during the first cycle. (b and c) S 2p XPS spectra of the pristine $Li_2S$ cathodes with Co@MX and without Co@MX.

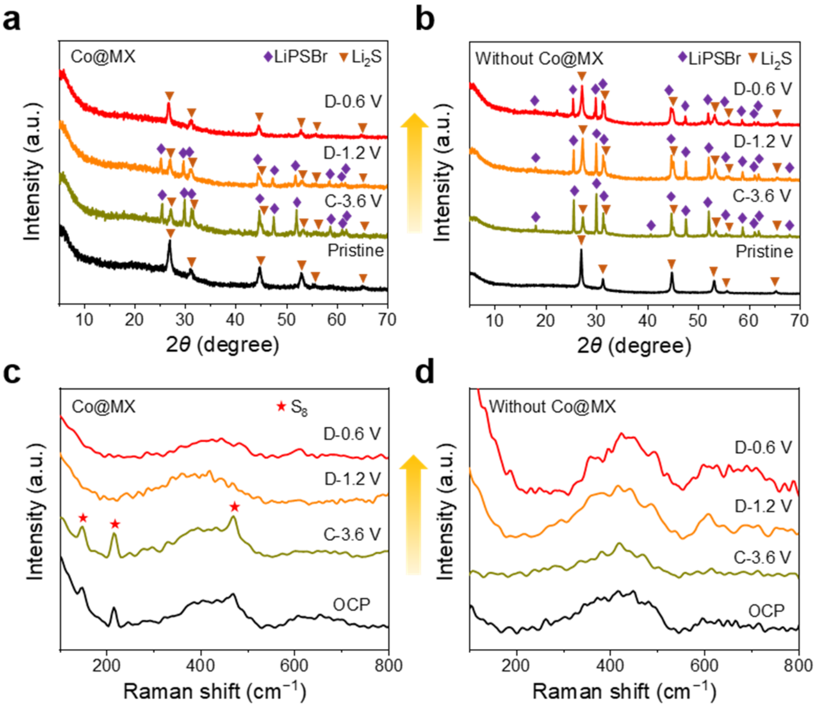


**Fig. S14** (a and b) *Ex situ* XRD patterns and (c and d) *ex situ* Raman spectra of the cathodes with/without Co@MX during the first cycle.

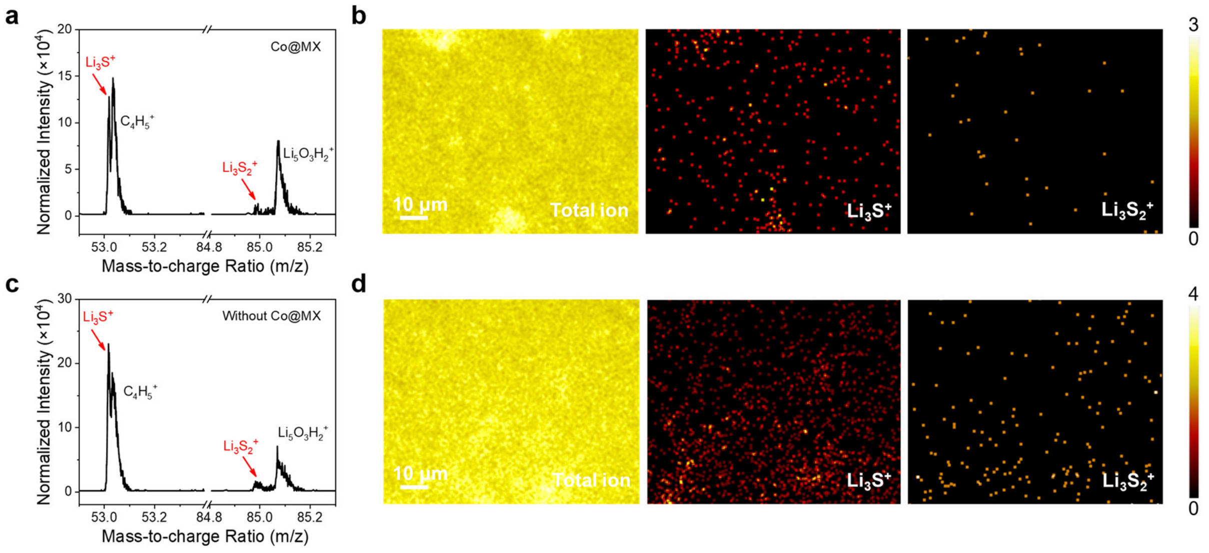


**Fig. S15** (a and c) TOF-SIMS spectra in the mass ranges of $Li_3S^+$ and $Li_3S_2^+$ of the discharge products at D-0.6 V for the cathodes with Co@MX and without Co@MX. The spectra are normalized to their total ion intensity for comparison. (b and d) TOF-SIMS ion distribution graphs of $Li_3S^+$ and $Li_3S_2^+$ for the cathodes with Co@MX and without Co@MX at D-0.6 V.

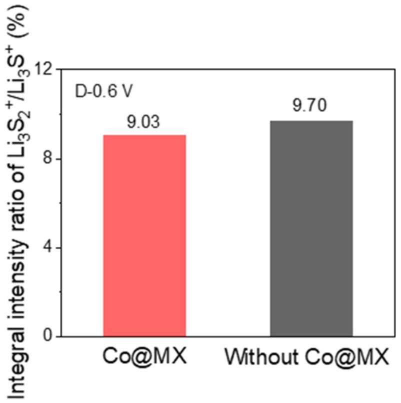


**Fig. S16** The $Li_3S_2^+/Li_3S^+$ integral ion intensity ratio obtained from TOF-SIMS for the cathodes with/without Co@MX at D-0.6 V. The Co@MX-based cathode demonstrates a relatively lower integral ion intensity ratio of $Li_3S_2^+/Li_3S^+$, illustrating the enhanced $Li_2S_2$-to-$Li_2S$ reduction reaction by Co@MX.

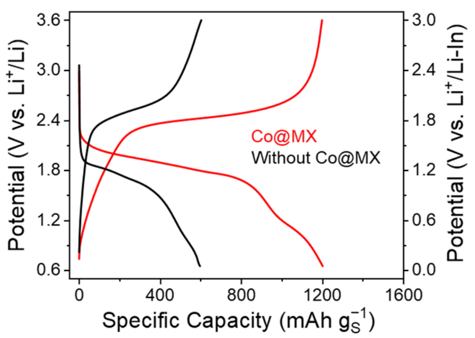


**Fig. S17** Galvanostatic charge–discharge profiles of ASSLSBs with/without Co@MX with a $Li_2S$ loading of 1.1 mg cm$^{-2}$ at 0.4 mA cm$^{-2}$.

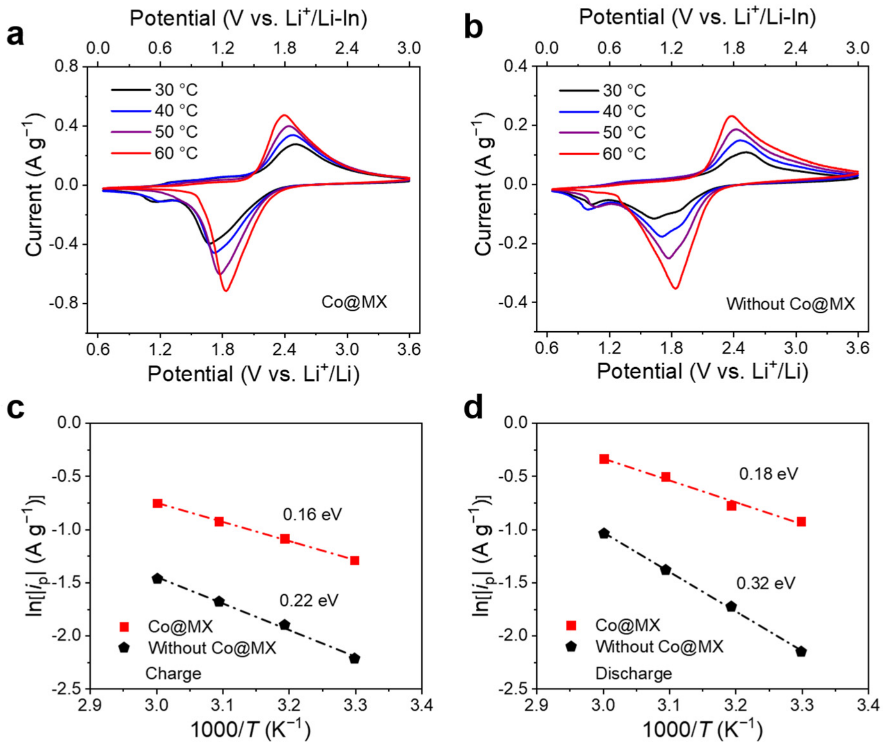


**Fig. S18** (a and b) CV profiles of ASSLSBs with Co@MX and without Co@MX at different temperatures with a scan rate of 0.1 mV s$^{-1}$. (c and d) Apparent activation energies of the charge and discharge process for ASSLSBs with/without Co@MX obtained from CV profiles at different temperatures.

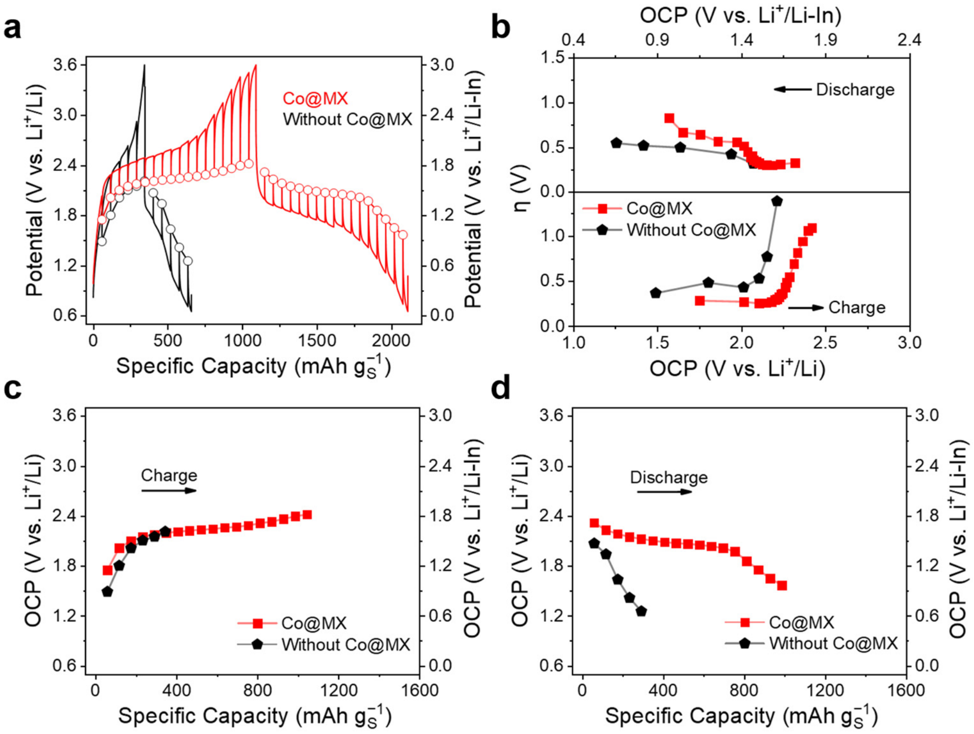


**Fig. S19** (a) GITT profiles of ASSLSBs with/without Co@MX. Current pulses of 0.08 A $g^{-1}$ for 30 min were applied followed by a 4 h relaxation. (b) Overpotential profiles obtained from the GITT measurement. (c and d) Open-circuit potential (OCP) profiles for the charge and discharge process. Assuming that the electrochemical reactions in both cathodes have reached thermodynamic equilibrium after each 4 h resting at OCP, the capacity of the Co@MX-based cathode overall outperforms that without Co@MX especially for the discharge process at the same OCP, which corroborates the sufficient electrochemically active sulfur in the Co@MX-based cathode [10].

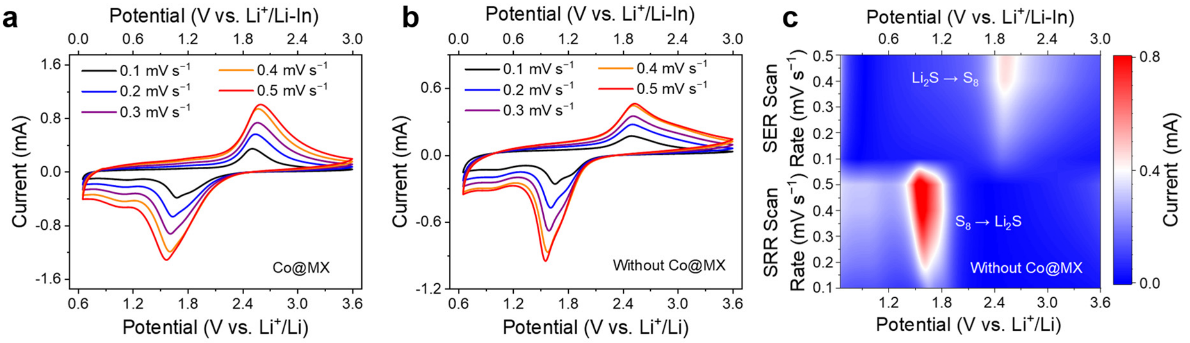


**Fig. S20** (a and b) CV profiles of ASSLSBs with Co@MX and without Co@MX at different scanning rates (from 0.1 to 0.5 mV s$^{-1}$). (c) Contour plots of CV profiles for the cathode without Co@MX.

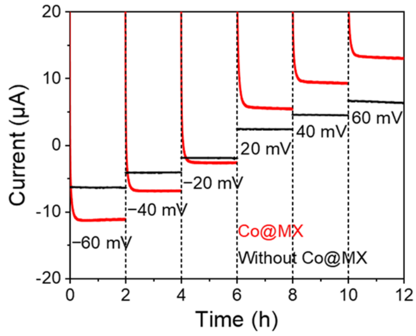


**Fig. S21** DC polarization measurements under electronic blocking condition for the cathode composites with/without Co@MX.

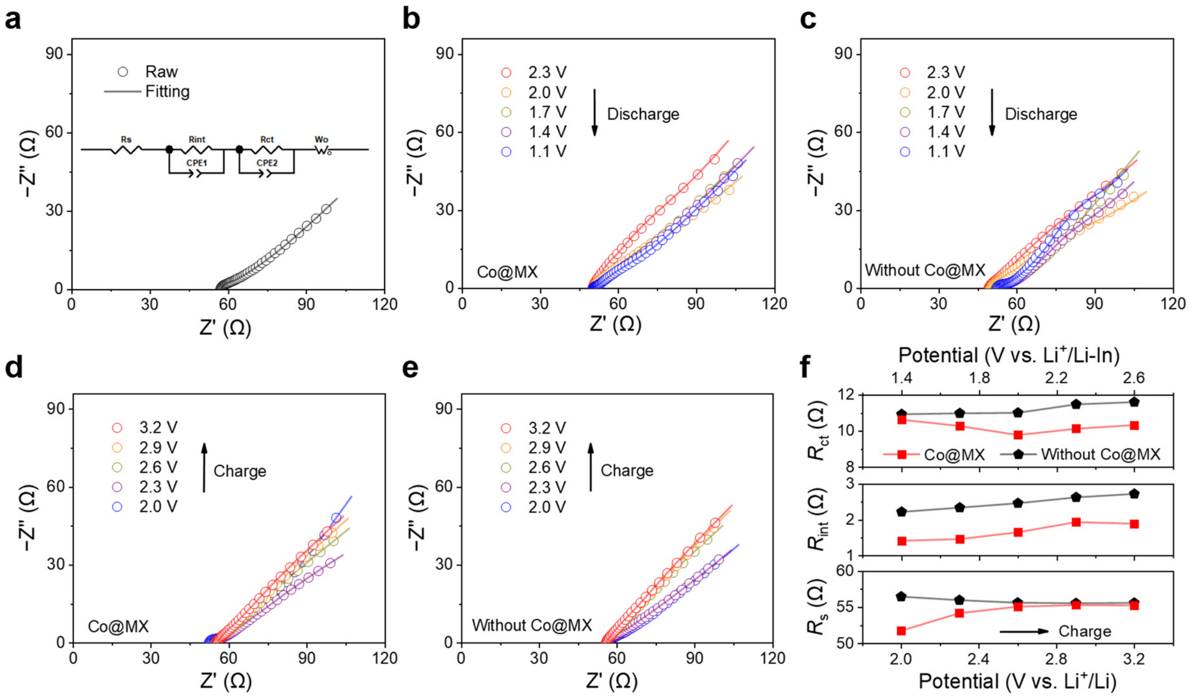


**Fig. S22** (a) Equivalent circuit model for fitting the EIS spectra. The inset is the equivalent circuit model. (b–e) Nyquist plots of *in situ* EIS measurement for the discharge process with Co@MX and without Co@MX, and for the charge process with Co@MX and without Co@MX. (f) Evolution of $R_s$, $R_{int}$, and $R_{ct}$ for ASSLSBs with/without Co@MX at different charge potentials revealed by *in situ* EIS measurement.

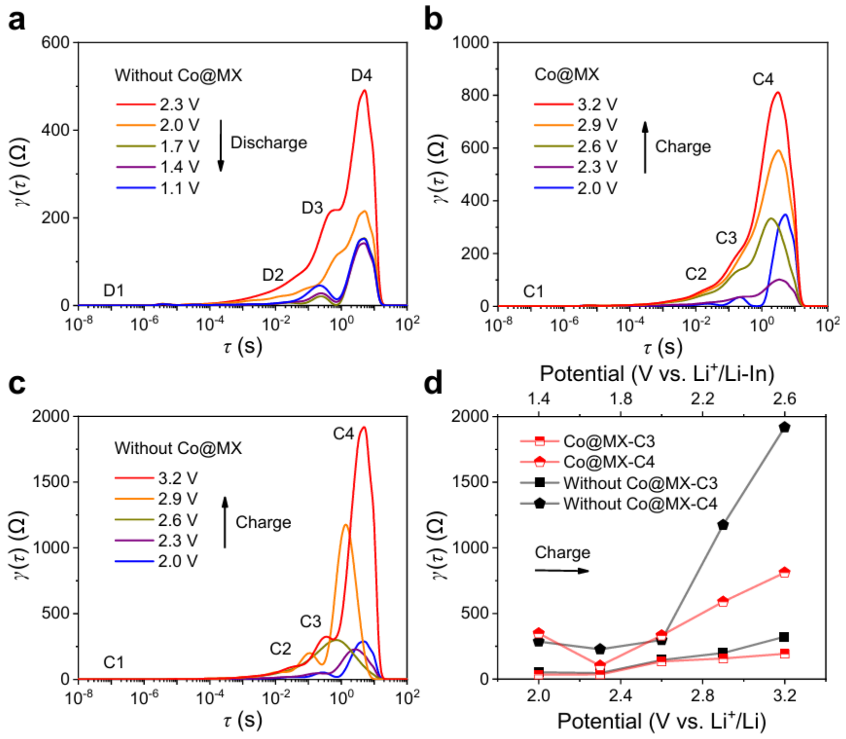


**Fig. S23** (a–c) DRT curves calculated from *in situ* EIS measurement of ASSLSBs for the discharge process without Co@MX, and for the charge process with Co@MX and without Co@MX. (d) The relaxation-based function $\gamma(\tau)$ obtained from DRT spectra for the charge process with/without Co@MX.

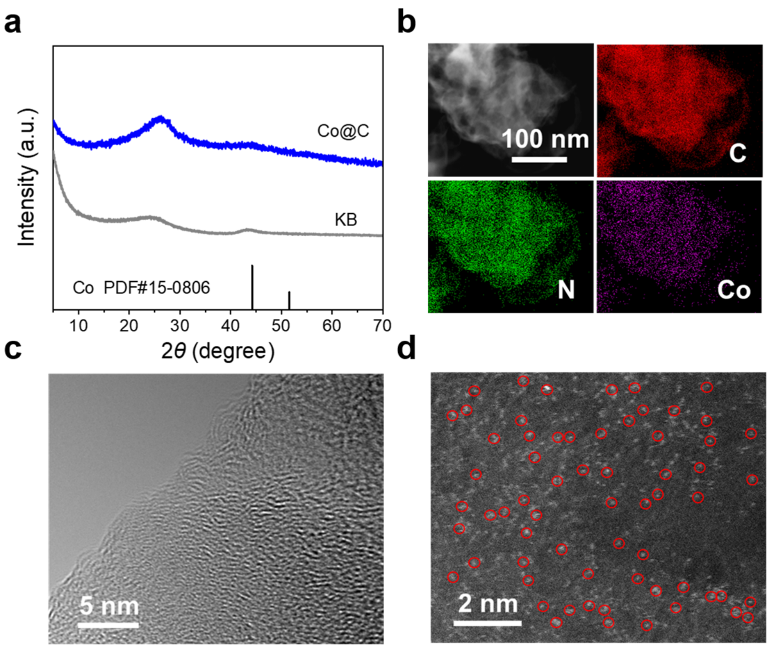


**Fig. S24** (a) XRD patterns of Co@C and KB. (b) EDS elemental mapping images, (c) TEM image and (d) HAADF-STEM image of Co@C. These results confirm the existence of Co SAs on porous carbon substrate.

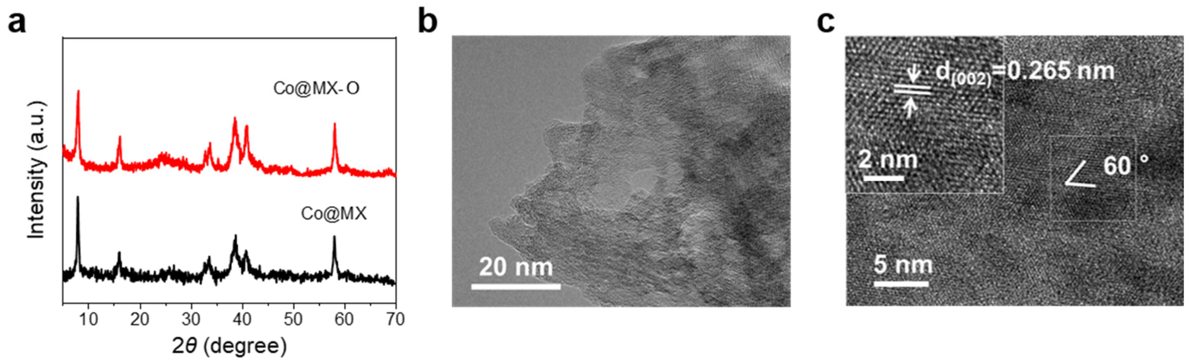


**Fig. S25** (a) XRD patterns of Co@MX-O. (b and c) TEM images of Co@MX-O. Since the conventional HF acid etching method cannot introduce Co SAs on MXene-O, we prepared Co SAs on O-rich MXene by immersing Co@MX in KOH solution followed by centrifugation-rinsing cycles with DI water. After drying, the product was obtained, defined as Co@MX-O. XRD and TEM results reveal that Co@MX-O preserves the structural integrity similar to its precursor (Co@MX) after alkali treatment. High-resolution TEM image shows the lattice spacing of (002) plane of Co@MX-O is ~0.26 nm, identical to that of Co@MX (Fig. S2).

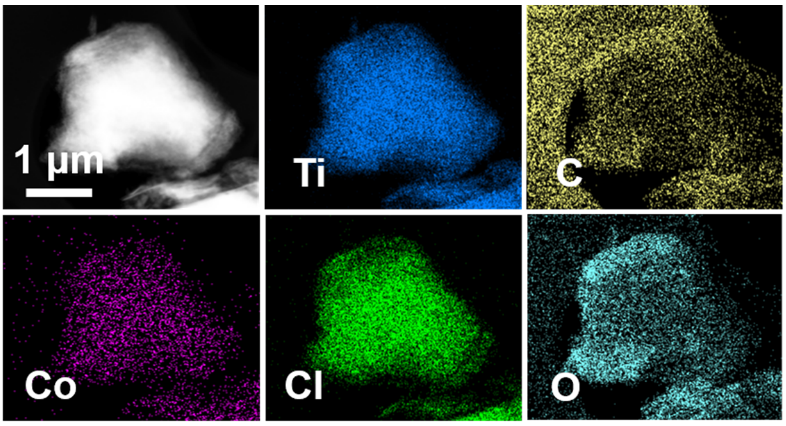


**Fig. S26** TEM image and corresponding EDS elemental mapping images of Co@MX-O. The EDS elemental mapping images of Co@MX-O show a significant increase in O content and a concurrent reduction in Cl content (The atom ratio of O to Cl is 1.55:1) compared to those of Co@MX (The atom ratio of O to Cl is 0.32:1) (Fig. S27, Table S3 and S4). Notably, the Co loading nearly unchanged. These results together could collectively confirm the existence of Co SAs and the introduction of O functional groups on the MXene surface.

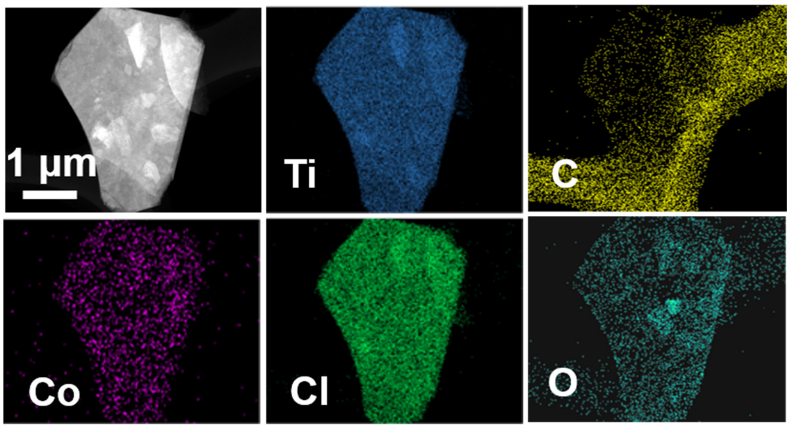


**Fig. S27** TEM image and corresponding EDS elemental mapping images of Co@MX.

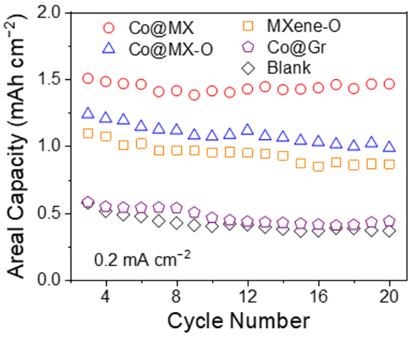


**Fig. S28** Cycling performance of ASSLSBs using different catalysts with a $Li_2S$ loading of 2.4 mg cm$^{-2}$ at 0.2 mA cm$^{-2}$. The battery performance of the Co@MX-O-based cathode is between Co@MX and MXene-O. Moreover, the discharge capacity and cycling stability of the cathode with Co@MX keep higher than those of Co@MX-O. The performance comparison suggests the synergistic effect of Co@MX in which Co SAs contribute to facile S-S bond cleavage and Cl-rich MXene is beneficial to rapid $Li^+$ diffusion.

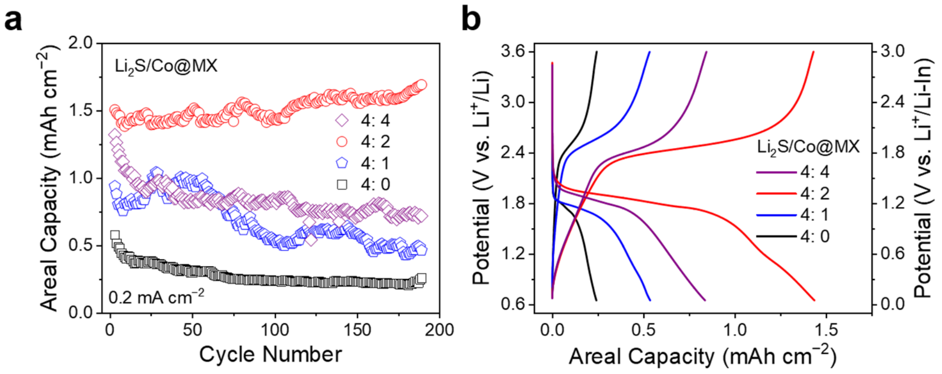


**Fig. S29** (a and b) Cycling performance and galvanostatic charge–discharge profiles at the 100th cycle of ASSLSBs with different mass ratios of $Li_2S$ to Co@MX. The $Li_2S$ loading is ~2.5 mg cm$^{-2}$ and the current density is 0.2 mA cm$^{-2}$.

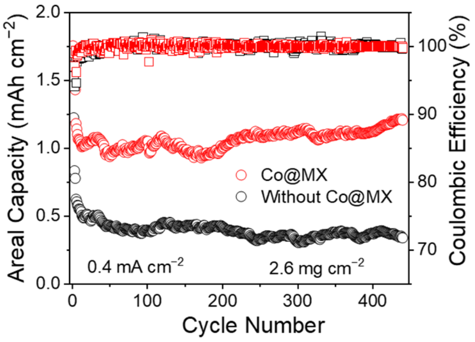


**Fig. S30** Cycling performance of ASSLSBs with/without Co@MX with a $Li_2S$ loading of 2.6 mg cm$^{-2}$ at 0.4 mA cm$^{-2}$.

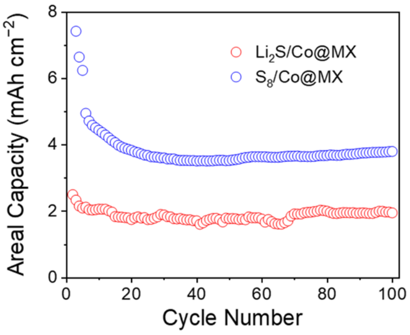


**Fig. S31** Cycling performance of Co@MX-based high-loading ASSLSBs with a $Li_2S$ loading of 5.2 mg cm$^{-2}$ at 0.1 mA cm$^{-2}$, and $S_8$ loading of 2.9 mg cm$^{-2}$ at 0.8 mA cm$^{-2}$.

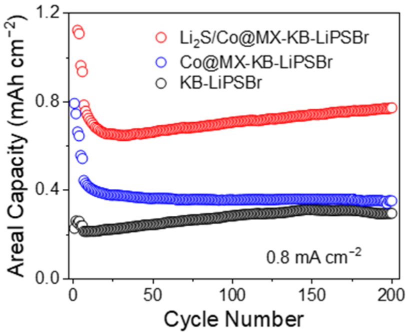


**Fig. S32** Cycling performance of the Co@MX-based sulfur cathode, Co@MX-KB-LiPSBr cathode and sole KB-LiPSBr with a LiPSBr loading of 1.1 mg cm$^{-2}$ at 0.8 mA cm$^{-2}$. The Co@MX-KB-LiPSBr cathode delivers a slightly higher capacity than sole KB-LiPSBr cathode after 200 cycles, which could be ignored over prolonged cycling. Hence, Co@MX itself yields a limited even negligible capacity contribution for ASSLSBs. We have mentioned that reversible lithiation/delithiation of LiPSBr could cause some extra capacity, as observed in the performance of sole KB-LiPSBr cathode. However, the actual capacity contribution from LiPSBr decomposition in operation is substantially lower, since the contact and reaction between LiPSBr and KB will be reduced in the presence of $Li_2S$ and Co@MX. Hence, the markedly high and gradually increasing battery capacity is primarily attributed to the efficient utilization of $Li_2S/S_8$ active materials, facilitated by Co@MX catalyst.

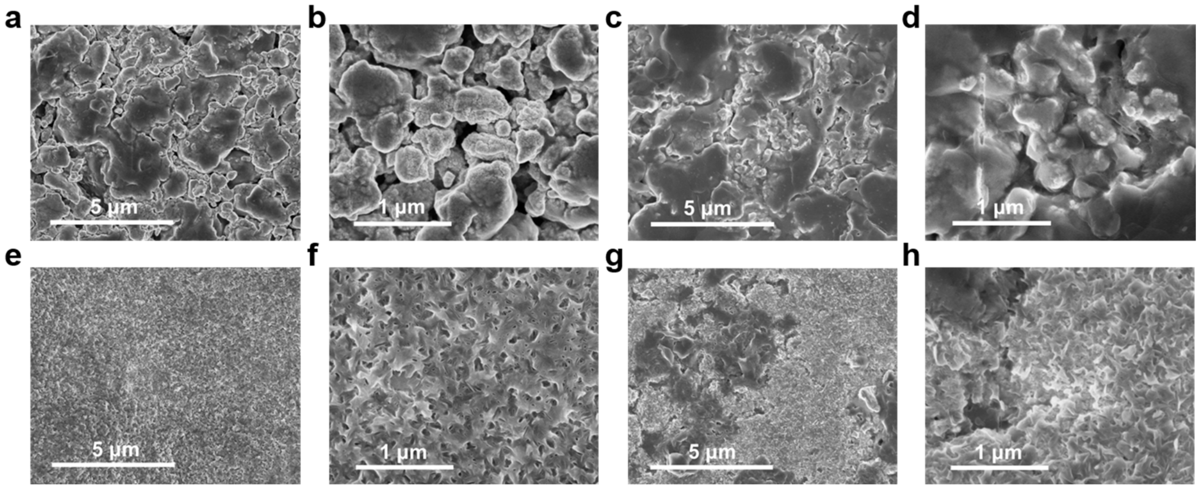


**Fig. S33** SEM images of the Co@MX-based cathode (a and b) before cycling and (e and f) after 100 cycles. SEM images of the cathode without Co@MX (c and d) before cycling and (g and h) after 100 cycles. The initial micron-sized $Li_2S$ particles underwent obvious pulverization and the fragmented $Li_2S$ were uniformly distributed in the Co@MX-based composite cathode after cycling. Compared to the LiPSBr electrolyte evenly coated by abundant pulverized $Li_2S$, there were still large LiPSBr or $Li_2S$ particles exposed in the cathode without Co@MX.

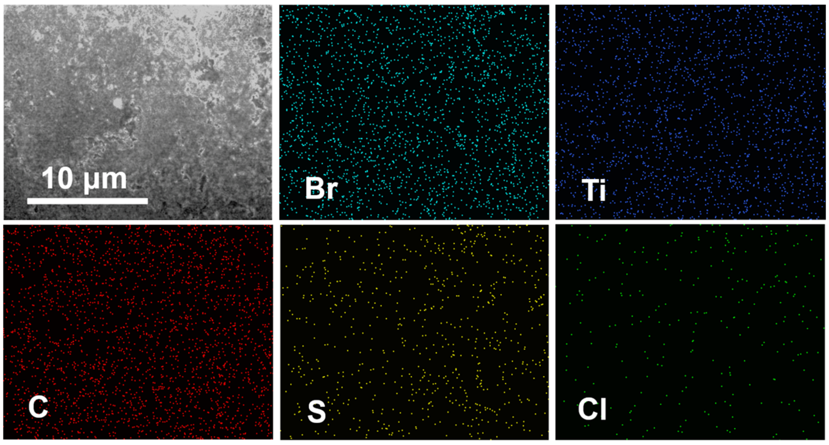


**Fig. S34** SEM image and corresponding EDS elemental mapping images of the Co@MX-based cathode after 100 cycles. Assisted by the Co@MX matrix, a large amount of active material experienced pulverization and reaction after cycling. The resulting fragments homogeneously mixed with the LiPSBr electrolyte and maintained good contact, ensuring continuous $Li^+$ conduction and full conversion.

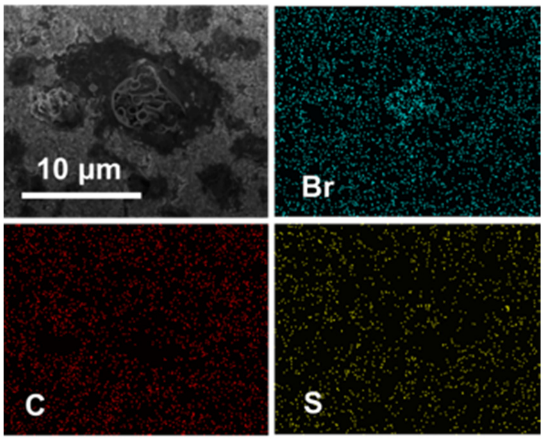


**Fig. S35** SEM image and corresponding EDS elemental mapping images of the cathode without Co@MX after 100 cycles. In the absence of Co@MX, the large active material particles and exposed electrolytes led to an uneven and insufficient reaction.

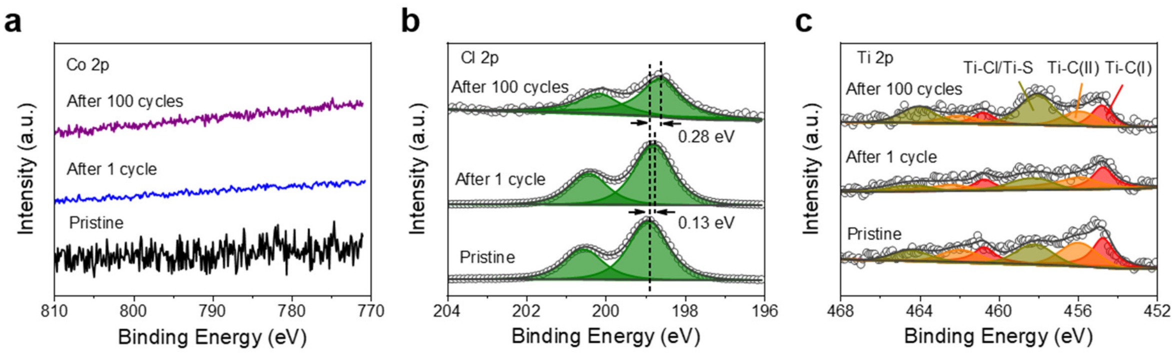


**Fig. S36** (a) Co 2p, (b) Cl 2p and (c) Ti 2p XPS spectra of the Co@MX-based sulfur cathode before and after cycling. Although the Co content was too low to be detected by XPS, the Cl 2p peaks of Co@MX shifted by 0.28 eV toward lower binding energy after 100 cycles, attributed to the electron-withdrawing effect of surface Cl-groups of Co@MX. Meanwhile, in the Ti 2p spectra, the intensity of Ti-Cl/Ti-S peaks increased (the two Ti 2p peak positions are very close [11,12]), which is likely due to the enhanced interaction between sulfur and exposed Ti atoms at Co@MX surface defects. These changes indicate that more sulfur species interact with Co@MX catalyst during long cycling and prove the progressively improved utilization of Co@MX active sites.

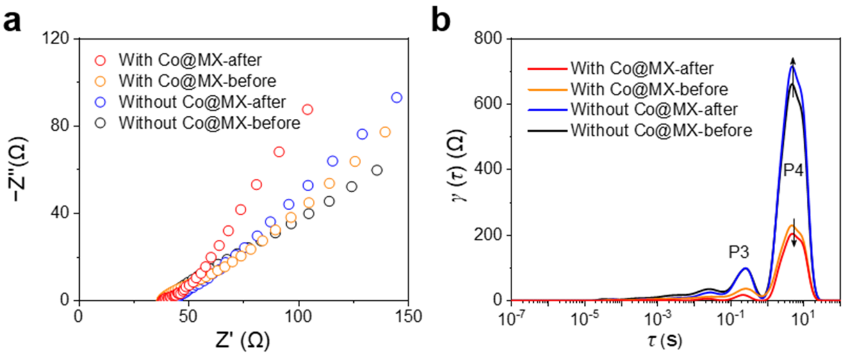


**Fig. S37** (a) Nyquist plots of the EIS measurement and (b) DRT curves calculated from the EIS measurement of ASSLSBs for the cathodes with/without Co@MX before and after cycling. From DRT analysis, we distinguished the charge transfer resistance at the cathode interface (labelled as P3 peak) and within the cathode (P4). The intensity of both P3 and P4 of Co@MX-based cathodes are much lower than those without Co@MX regardless of cycling. More importantly, we found that the resistance of P3 and P4 decreased after 100 cycles enabled by Co@MX. By contrast, the charge transfer resistance of the cathode without Co@MX significantly increased. This difference further validates that the activated Co@MX catalyst effectively facilitates the cathode charge transfer and the active material conversion.

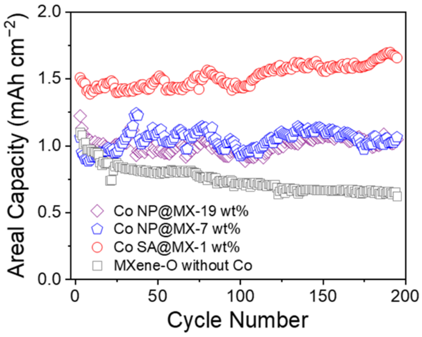


**Fig. S38** Cycling performance of MXene based ASSLSBs with different Co contents with a $Li_2S$ loading of 2.4 mg cm$^{-2}$ at 0.2 mA cm$^{-2}$.

**Table S1** Summary of the fitted data and error in Fig. 4f.

| Potential (V vs. $Li^+/Li$) | Parameter | **Without Co@MX** | | **Co@MX** | |
|---|---|---|---|---|---|
| | | Fitting value (Ω) | Error (%) | Fitting value (Ω) | Error (%) |
| 2.3 | $R_s$ | 48.77 | 0.09 | 49.75 | 0.13 |
| | $R_{int}$ | 2.22 | 3.87 | 1.82 | 5.92 |
| | $R_{ct}$ | 10.24 | 2.34 | 9.97 | 2.69 |
| 2.0 | $R_s$ | 49.87 | 0.14 | 50.05 | 0.11 |
| | $R_{int}$ | 3.09 | 3.91 | 2.00 | 4.16 |
| | $R_{ct}$ | 14.64 | 2.04 | 10.00 | 2.04 |
| 1.7 | $R_s$ | 51.84 | 0.13 | 50.18 | 0.05 |
| | $R_{int}$ | 3.12 | 3.18 | 2.04 | 2.05 |
| | $R_{ct}$ | 16.10 | 1.98 | 10.09 | 0.80 |
| 1.4 | $R_s$ | 52.13 | 0.12 | 50.35 | 0.05 |
| | $R_{int}$ | 4.58 | 5.36 | 2.07 | 2.34 |
| | $R_{ct}$ | 18.35 | 1.96 | 10.07 | 0.85 |
| 1.1 | $R_s$ | 52.39 | 0.17 | 50.33 | 0.05 |
| | $R_{int}$ | 4.93 | 5.77 | 2.10 | 2.38 |
| | $R_{ct}$ | 18.54 | 3.78 | 10.06 | 0.77 |

**Table S2** Summary of the fitted data and error in Fig. S22f.

| Potential (V vs. $Li^+$/Li) | Parameter | **Without Co@MX** | | **Co@MX** | |
|---|---|---|---|---|---|
| | | Fitting value (Ω) | Error (%) | Fitting value (Ω) | Error (%) |
| 2.0 | $R_s$ | 56.47 | 0.07 | 51.76 | 0.06 |
| | $R_{int}$ | 2.22 | 3.10 | 1.42 | 2.71 |
| | $R_{ct}$ | 10.93 | 1.86 | 10.63 | 0.96 |
| 2.3 | $R_s$ | 55.98 | 0.07 | 54.19 | 0.05 |
| | $R_{int}$ | 2.34 | 3.01 | 1.46 | 2.14 |
| | $R_{ct}$ | 10.98 | 1.75 | 10.28 | 2.19 |
| 2.6 | $R_s$ | 55.63 | 0.08 | 55.09 | 0.08 |
| | $R_{int}$ | 2.46 | 3.87 | 1.65 | 4.56 |
| | $R_{ct}$ | 11.01 | 2.45 | 9.78 | 2.60 |
| 2.9 | $R_s$ | 55.55 | 0.06 | 55.30 | 0.08 |
| | $R_{int}$ | 2.62 | 3.28 | 1.94 | 3.69 |
| | $R_{ct}$ | 11.48 | 2.37 | 10.13 | 2.29 |
| 3.2 | $R_s$ | 55.60 | 0.07 | 55.27 | 0.08 |
| | $R_{int}$ | 2.72 | 2.97 | 1.89 | 4.15 |
| | $R_{ct}$ | 11.60 | 2.20 | 10.33 | 2.62 |

**Table S3** Summary of the element contents of Fig. S26.

| Element | wt% | at% |
|---|---|---|
| Ti | 45.33 | 21.55 |
| C | 28.86 | 54.68 |
| **Co** | **1.88** | **0.73** |
| **Cl** | **14.08** | **9.04** |
| **O** | **9.85** | **14.00** |
| total | 100.00 | 100.00 |

**Table S4** Summary of the element contents of Fig. S27.

| Element | wt% | at% |
|---|---|---|
| Ti | 50.29 | 28.16 |
| C | 21.47 | 47.92 |
| **Co** | **1.69** | **0.77** |
| **Cl** | **23.20** | **17.54** |
| **O** | **3.35** | **5.61** |
| total | 100.00 | 100.00 |

## REFERENCES


1. Hua W, Shang T, Li H *et al.* Optimizing the *p* charge of S in *p*-block metal sulfides for sulfur reduction electrocatalysis. *Nat Catal* 2023; **6**: 174–84.
2. Kresse G, Furthmuller J. Efficient iterative schemes for ab initio total-energy calculations using a plane-wave basis set. *Phys Rev B Condens Matter* 1996; **54**: 11169–86.
3. Grimme S, Ehrlich S, Goerigk L. Effect of the damping function in dispersion corrected density functional theory. *J Comput Chem* 2011; **32**: 1456–65.
4. Blöchl PE. Projector augmented-wave method. *Phys Rev B* 1994; **50**: 17953.
5. Grimme S, Antony J, Ehrlich S *et al.* A consistent and accurate ab initio parametrization of density functional dispersion correction (DFT-D) for the 94 elements H-Pu. *J Chem Phys* 2010; **132**: 154104.
6. Wang V, Xu N, Liu J-C *et al.* VASPKIT: a user-friendly interface facilitating high-throughput computing and analysis using VASP code. *Comput Phys Commun* 2021; **267**: 108033.
7. Momma K, Izumi F. VESTA 3 for three-dimensional visualization of crystal, volumetric and morphology data. *J Appl Crystallogr* 2011; **44**: 1272–76.
8. Maintz S, Deringer VL, Tchougréeff AL *et al.* LOBSTER: a tool to extract chemical bonding from plane-wave based DFT. *J Comput Chem* 2016; **37**: 1030.
9. Henkelman G, Uberuaga BP, Jónsson H. A climbing image nudged elastic band method for finding saddle points and minimum energy paths. *J Chem Phys* 2000; **113**: 9901–4.
10. Wang D, Jhang L-J, Kou R *et al.* Realizing high-capacity all-solid-state lithium-sulfur batteries using a low-density inorganic solid-state electrolyte. *Nat Commun* 2023; **14**: 1895.
11. Li M, Lu J, Luo K *et al.* Element replacement approach by reaction with Lewis acidic molten salts to synthesize nanolaminated MAX phases and MXenes. *J Am Chem Soc* 2019; **141**: 4730–7.
12. Li Q, Xie C, Jiang X *et al.* Catalytic solder fuses solid-solid interfaces for all-solid-state lithium-sulfur batteries. *Adv Mater* 2025; 2507308.
