# Supplementary material for: Exploiting deep sulfur conversion by tandem catalysis for all-solid-state lithium–sulfur batteries
Source: Natl Sci Rev. 2025 Nov 21;13(1):nwaf525. doi: 10.1093/nsr/nwaf525 (PMC12796810; doi:10.1093/nsr/nwaf525)
Supplement: nwaf525_Supplemental_Files [file nwaf525_supplemental_files.zip › Teaser text.docx]

This work proposes tandem catalysis to exploit deep conversion of S_8_ to Li_2_S via intermediate Li_2_S_2_, achieving high-capacity all-solid-state lithium–sulfur batteries and deepening the understanding for solid-state catalytic mechanism.
